# Probability and Rate of Reinforcement in Negative Prediction Error Learning

**DOI:** 10.1037/xan0000396

**Published:** 2025-05-12

**Authors:** David J. Sanderson, Joseph M. Austen, Anthony McGregor, Jasmin A. Strickland

**Affiliations:** 1 Department of Psychology, Durham University

**Keywords:** learning, conditioned responding, trial, time, mice

## Abstract

Trial-based theories of associative learning propose that learning is sensitive to the probability of reinforcement signaled by a conditioned stimulus (CS). Learning, however, is often sensitive to reinforcement rate rather than probability of reinforcement per trial, suggesting that temporal properties of cues may be more important than trial-based properties. In four experiments, the role of probability of reinforcement per trial was examined in appetitive Pavlovian conditioning in mice under conditions in which reinforcement rate was controlled. Experiments 1 and 2 examined the loss of conditioned responding caused by overexpectation of reinforcement. The probability of reinforcement per trial failed to affect acquisition and summation of conditioned responding and failed to affect overexpectation. It also failed to affect extinction of conditioned responding in Experiments 3 and 4. Experiments 2–4 contained nonreinforced trials in which responding at the offset of the CS could be measured. These probe trials did reveal an effect of probability of reinforcement per trial. Cues associated with 100% reinforcement elicited greater post-CS responding than cues associated with 50% reinforcement. The effect was also evident in summation trials (in Experiment 2) in which two 100% or 50% reinforced cues were presented in compound. The results show that mice learn about rate and probability information, but reinforcement rate determines anticipatory responding during the CS. The probability of reinforcement determines responding at the expected time of reinforcement. Thus, learning occurs continuously over the duration of experience and per episode of experience independent of duration.

A consequence of describing changes in associative strength between a conditioned stimulus (CS) and an unconditioned stimulus (US) in a trial-by-trial manner is that associative strength is predicted to be proportional to the probability of reinforcement signaled by a cue. An alternative approach is to describe changes in learning that happen iteratively over some constant unit of time such that the process is continuous rather than occurring in discrete events of potentially differing durations. This results in learning being updated not only during the periods of reinforcement but also in the periods of nonreinforcement during a CS prior to the occurrence of the US. Therefore, associative strength will be influenced by the reinforcement rate of the CS rather than the probability of reinforcement per trial. Indeed, cues may differ in their predicted probability of reinforcement per trial, but if they are matched for overall rate of reinforcement, then learning should proceed similarly (Harris et al., 2015).

Research in appetitive Pavlovian magazine approach conditioning has shown that rats learn about both (i) the reinforcement rate of cues over cumulative exposure, independent of how the exposure is structured in terms of trials, and (ii) the probability of reinforcement per trial, independent of reinforcement rate. Evidence for learning being determined by reinforcement rate rather than probability of reinforcement per trial has been shown in the acquisition of conditioned responding. Harris et al. (2015) found that, over a wide range of parameters, there was no effect of partial reinforcement on acquisition of conditioned responding when cues were matched for their overall reinforcement rate by manipulation of trial durations. For example, a cue that was on average 10 s long and reinforced on a third of its trials elicited levels of conditioned responding over acquisition similar to a cue that was on average 30 s long and reinforced on every trial. Furthermore, when probability of reinforcement per trial is held constant but reinforcement rate is manipulated by differences in the average trial duration, the strength of conditioned responding increases as a function of reinforcement rate (Harris & Carpenter, 2011). This pattern of results has also been found in magazine approach behavior in mice (Austen et al., 2021; Austen & Sanderson, 2019; Strickland et al., 2024).

Evidence for a role of probability of reinforcement per trial comes from research on the partial reinforcement extinction effect (PREE) in which conditioned responding reduces more slowly during extinction learning for a previously partially reinforced cue than a previously continuously reinforced cue (Amsel, 1962; Capaldi, 1966). Chan and Harris (2019) found the PREE occurred in Pavlovian conditioning for cues that were matched for their previous reinforcement rate. Therefore, slower extinction for partially reinforced cues could not be attributed to rate-based explanations of the effect (see Gallistel & Gibbon, 2000) but reflected solely the effect of probability of reinforcement per trial.

Although the probability of reinforcement per trial does not affect acquisition of conditioned responding during a CS, it does affect responding in the moments immediately after the termination of a CS. Harris and Kwok (2018) examined acquisition with two partially reinforced cues that differed in their probability of reinforcement per trial. Post-CS responding was examined on the nonreinforced trials in the absence of reinforcement. Rats showed greater immediate posttrial responding to a cue that had a higher probability of reinforcement compared to a cue with a lower probability. Furthermore, when cues differed in the rate of reinforcement but were matched in probability of reinforcement per trial, post-CS responding was similar.

The dissociation between reinforcement rate and probability of reinforcement per trial suggests that trial-based learning determines responding when the US is omitted (Harris, 2019). During acquisition, this effect is limited to post-CS responding. Thus, it does not affect the rate at which animals respond during the CS. However, in extinction learning when the overall contingency between the CS and US is altered, the probability of reinforcement per trial determines the rate at which responding extinguishes. Therefore, the probability of reinforcement may determine the extent of learning as a consequence of negative prediction error. If probability of reinforcement per trial determines negative prediction error generally, then it should affect the losses of conditioned responding when it occurs not only in the absence of reinforcement, as in extinction learning, but also in the presence of reinforcement, as in overexpectation of reinforcement (e.g., Kremer, 1978). Thus, negative prediction error will occur when the US is expected but the CS is nonreinforced and also when a CS is reinforced but the amount of reinforcement is less than expected (i.e., overexpectation).

The purpose of the present experiments was to test the circumstances under which the probability of reinforcement affects learning. Learning was assessed in Pavlovian appetitive magazine approach conditioning in female mice. In all of the experiments, learning was tested with cues that differed in probability of reinforcement but were matched for rate of reinforcement. Experiments 1 and 2 examined the effect of probability of reinforcement on overexpectation in which negative prediction occurs in the presence of reinforcement. Experiments 3 and 4 examined extinction learning in the absence of reinforcement.

## Experiment 1

Experiment 1 tested the role of probability of reinforcement per trial in overexpectation. Overexpectation occurs when two previously reinforced CSs (A+ and X+) are presented in compound and reinforced to the same extent as the previous individual CS presentations (AX+). At test, the compound conditioning results in a loss of conditioned responding to the individual cues. The Rescorla and Wagner (1972) model accounts for the loss of conditioned responding by proposing that the associative strength of the individual cues is summed when first presented in compound. Because the summed associative strength of the compound is greater than the associative strength supported by the level of reinforcement presented, the associative strength of the individual cues is reduced over the course of compound training. In other words, when initially presented with the compound, animals expect a level of reinforcement that is greater than is presented.

The design of Experiment 1 is shown in Table 1. Overexpectation was tested in a within-subjects design. The manipulation of probability of reinforcement per trial, however, was between subjects. Mice were trained with four cues, A, X, B, and Y. Cues A and X were used to test overexpectation. For mice in Group 100%, Cues A and X were on average 20 s in duration and reinforced on every trial. For Group 50%, Cues A and X were on average 10 s in duration and reinforced on 50% of trials. Therefore, although the groups differed in the probability of reinforcement per trial, because of the difference in average duration, the cumulative rate of reinforcement of A and X was matched across groups. In Stage 2, mice received trials in which A and X were presented in compound, reinforced on every trial and on average 20 s in duration. If overexpectation reflects an expectation of the summed amount of reinforcement per trial, then during Stage 2, there should be an overexpectation effect for Group 100%, but this effect will be attenuated or abolished for Group 50%. If associative strength corresponds linearly to probability of reinforcement then because A and X were both associated with reinforcement on every trial, Group 100% should expect twice as much reinforcement as was presented. In contrast, for Group 50%, because A and X were previously associated with reinforcement on 50% of trials (and thus on average 50% reinforcement per trial), when presented with AX, they should expect one amount of reinforcement per trial, and consequently, overexpectation will not occur. If, however, overexpectation reflects the summation of reinforcement rates because the rates of reinforcement were matched across Stage 1, both groups should show a similar overexpectation effect.[Table tbl1]

It is important to note that the Rescorla and Wagner (1972) model does not assume that associative strength corresponds linearly to the probability of reinforcement because it assumes that the learning rate in the presence of reinforcement is greater than in the absence of reinforcement. This assumption is necessary for the model to account for findings such as the relative validity effect (Wagner et al., 1968). In contrast, however, for the model to be able to account for the correspondence between reinforcement rate and response rate in compound conditioning (Harris et al., 2012), it has to be assumed that the learning rates are equal in the presence and absence of reinforcement. Given these issues, the hypothesis is that the manipulation of probability should, at the least, attenuate overexpectation if not fully abolish overexpectation.

Cues B and Y served as control cues for the overexpectation effect. Their treatment was the same for both groups. In Stage 1, Cue B was on average 20 s long and reinforced on 100% of trials. Cue Y was also on average 20 s long but nonreinforced. In Stage 2, in addition to AX compound trials, mice were presented with trials in which B and Y were presented in compound, reinforced on every trial and on average 20 s in duration. Because Cue B was associated with reinforcement on every trial and Cue Y was associated with nonreinforcement, when reinforced in compound on 100% of trials, there will be no overexpectation. The crucial comparison for overexpectation was between Cues A and B. Whereas responding should reduce for A as a consequence of the compound training in Stage 2, responding for B should not.

Various control methods for overexpectation have been used that range from methods similar to those used here to procedures such as continued reinforced training of a cue not presented in compound during Stage 2 or no training at all during Stage 2 (Arico & McNally, 2014; Garfield & McNally, 2009; Haney et al., 2010; Holland, 2016; Iordanova et al., 2016; Kehoe & White, 2004; Khallad & Moore, 1996; Kremer, 1978; Lattal & Nakajima, 1998; Lay et al., 2019, 2023a, 2023b; Lucantonio et al., 2015; McNally et al., 2004; Rescorla, 1970, 1999, 2006, 2007; Ruprecht et al., 2014; Sissons & Miller, 2009; Terao et al., 2022; Witnauer & Miller, 2009). The advantage of the use of Cues B and Y as a within-subjects control procedure for overexpectation is that it allows various explanations for the reduced responding to A be ruled out. Because the procedure is within subjects, differences between responding to A and B cannot be attributed to general effects on response levels, which may occur in between-subjects designs. Furthermore, certainly for Group 100%, A and B are matched for manner of presentation and reinforcement contingency and pairings across Stages 1 and 2. Therefore, differences in responding to A and B during the test phase cannot be attributed to differences in exposure in the previous stages that may, subsequently, result in differences in generalization decrement. Each aspect of the procedure designed to result in overexpectation for Cue A is matched for Cue B except for the overexpectation of reinforcement.

Cue durations were variable over trials. Variable durations reduce the influence of reinforcement timing on response rates within trials (Harris & Carpenter, 2011). Harris et al. (2015) showed that response rates are sensitive to reinforcement rates, independent of trial durations and probability of reinforcement, when animals are unable to time the occurrence of reinforcement. In addition to these factors, variable duration cues were chosen for the current study to reduce any generalization decrement that may occur when the average duration of cues is changed between Stages 1 and 2 for Group 50%.

### Method

#### Subjects

Thirty-two naïve female C57BL/6J mice (Charles River UK Ltd), approximately 10 weeks old at the start of testing, with a mean free-feeding weight of 18.6 g (range = 16.4–20.3) were used. Mice were caged in groups of eight in a temperature-controlled housing room on a 12-hr light–dark cycle (lights on at 8:00 a.m.). Prior to the start of the experiment, the weights of the mice were reduced by being placed on a restricted diet. Mice were then maintained at 85% of their free-feeding weights throughout the experiment. Mice had ad libitum access to water in their home cages. All procedures were in accordance with the United Kingdom Animals Scientific Procedures Act (1986); under Project License P8B95F992.

#### Apparatus

A set of eight identical operant chambers (interior dimensions: 15.9 × 14.0 × 12.7 cm; ENV-307A, Med Associates, Inc., Fairfax, Vermont, United States) enclosed in sound-attenuating cubicles (ENV-022V) were used. The operant chambers were controlled by Med-PC IV software (SOF-735). The side walls were made from aluminum, and the front and back walls and the ceiling were made from clear Perspex. The chamber floors each comprised a grid of stainless steel rods (0.32-cm diameter), spaced 0.79 cm apart, and running perpendicular to the front of the chamber (ENV-307A-GFW). A food magazine (2.9 × 2.5 × 1.9 cm; ENV-303M) was situated in the center of one of the sidewalls of the chamber, into which sucrose pellets (14 mg, TestDiet) could be delivered from a pellet dispenser (ENV-203-14P). An infrared beam (ENV-303HDA) across the entrance of the magazine was used to record head entries at a resolution of 0.1 s. A fan (ENV-025F) was located within each of the sound-attenuating cubicles and was turned on during sessions, providing a background sound pressure level of approximately 65 dB. Auditory stimuli were provided by a white noise generator (ENV-325SM) outputting a flat frequency response from 10 to 25,000 Hz at 75 dB and a clicker (ENV-335M) operating at a frequency of 4 Hz at 75 dB. Visual stimuli were a 2.8 W house light (ENV-315M), which could illuminate the entire chamber, and two light emitting diodes (LEDs; ENV-321M) positioned to the left and right of the food magazine, which provided more localized illumination.

#### Procedure

Mice were randomly assigned to one of the two groups (*N* = 16 per group) that differed in their training during Stage 1. Group 50% received 12 sessions of training with two short duration cues, A and X, that were on average, across trials, 10 s (range = 2–18) in duration, and two other cues, B and Y, that were on average, across trials, 20 s (range = 2–38) in duration. The durations of the cues varied across trials according to a flat distribution with the constraint that the mean duration for A and X, and B and Y, was 10 and 20 s, respectively, within a session. Cues A and X were both reinforced on a random 50% of trials. Cue B was reinforced on 100% of trials, and Cue Y was nonreinforced. Cues were reinforced by the presentation of a sucrose pellet at the termination of the cue presentation. Group 100% received similar training except that Cues A and X were variable duration 20 s cues rather than 10 s and were reinforced on 100% of trials.

Within each session, Group 100% received three trials of each trial type and Group 50% received six trials of A and X each and three trials of B and Y each. Trial types were presented in a random order with the constraint that for Group 50%, each block of six trials consisted of two A trials, two X trials, one B trial, and one Y trial, and for Group 100%, each block of four trials consisted of one trial of each of the four different trial types. The difference in trial numbers for the two groups resulted in both groups receiving the same cumulative exposure to Cues A and X and the same number of pairings with reinforcement. In order to equate the session lengths, the fixed intertrial interval for Group 50% was 120 s (from CS offset to CS onset), and for Group 100%, it was 180 s. The sessions lasted 40 min.

Within both groups, for half of the mice, Cues A and B were auditory (white noise and clicker), and X and Y were visual (house light and alternating left and right flashing-LEDs, 0.5 s on, 0.5 s off). Within the subgroups allocated auditory A and B and visual X and Y cues, the allocation of individual stimuli to cue types was fully counterbalanced in a two-by-two design. For the other half of mice, A and B were visual and X and Y were auditory, and the allocation of stimuli to cue types was also fully counterbalanced in a two-by-two design.

In Stage 2, the groups received identical treatment. Mice received three sessions in which cues were now presented in the compounds AX and BY. Each compound was presented for a variable 20 s duration and was reinforced at the offset of the compound. Each compound was presented for nine trials per session. Trial types were presented in a random order with the constraint that each block of six trials contained three trials of each trial type. The intertrial interval was a fixed duration of 150 s. The sessions lasted 51 min.

In Stage 3, mice received two sessions that consisted of reinforced presentations of the compounds AX and BY and nonreinforced probe presentations of A and B. All trial types were a fixed duration of 20 s. Sessions consisted of six presentations of A and B each and three presentations of AX and BY each. Each block of six trials consisted of two trials of A and B each and one trial of AX and BY each. The order of trial types within a six-trial block was random with the constraint that within groups the order of A and B was counterbalanced with respect to the allocation of stimuli to trial types. The intertrial interval was a fixed duration of 150 s. The sessions lasted 51 min.

#### Data and Statistical Analysis

The frequency of head entries into the food magazine was recorded per-second during the CS presentations and the for the 10 s pre-CS period. The response rates are expressed as rate per minute (RPMs). Across the different stages, response rates were analyzed using multifactorial analyses of variance (ANOVAs) that included the factor of group and cue identity. Interactions were analyzed with simple main effects analysis using the pooled error term from the original ANOVA or separate ANOVAs for repeated measures with more than two levels. Where sphericity of within-subjects variables could not be assumed, the Greenhouse–Geisser correction was applied.

In instances in which statistical tests of key predictions failed to be significant, the strength of evidence for the null hypothesis was tested with Bayesian analyses carried out in JASP (JASP Team, 2024) using the default priors. For Bayesian ANOVAs, Bayes factors for the main effects or interactions on which the evidence for the hypothesis depends reflect comparison of models that include the main effect or interaction with similar models that exclude the main effect or interaction.

In order to simplify the analysis of pre-CS response rates, the analyses are collapsed across trial types because for all experiments, the order of trial types was either randomized within particular constraints or was counterbalanced across animals.

#### Transparency and Openness

The study was not preregistered. The data are available upon request. Sample sizes were approximately *N* = 16 per between-subjects condition. This number was chosen to ensure sufficient numbers for counterbalancing of multiple factors and to achieve 80% power for effects size approximately equal to η_p_^2^ = .20.

### Results and Discussion

#### Stage 1

**Figure 1 fig1:**
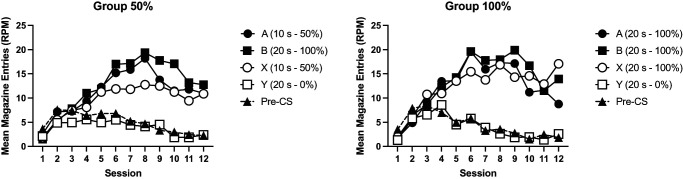
Acquisition of Conditioned Responding in Stage 1, Experiment 1 *Note.* Mean magazine entries are shown as RPM. Cues A, B, and X were reinforced and Cue Y was nonreinforced. For each cue, the durations and percentages indicate the mean trial duration and probability of reinforcement per trial. RPM = rate per minute; A = Cue A; B = Cue B; X = Cue X; Y = Cue Y; CS = conditioned stimulus.

Acquisition of responding is shown in Figure 1. Mice acquired conditioned responding to Cues A, B, and X. Responses to Y initially increased but then decreased over training. Response rates were similar between the two groups. A 4 (cue: A, B, X, and Y) by 12 (session) by 2 (group: 50%, 100%) ANOVA was conducted. There were significant effects of cue, *F*(3, 90) = 14.48, *p* < .001, η_p_^2^ = .33, 90% confidence interval (CI) = [.18, .42], session, *F*(11, 330) = 28.50, *p* < .001, η_p_^2^ = .49, 90% CI = [.41, .52], and a significant interaction of factors, *F*(33, 990) = 9.02, *p* < .001, η_p_^2^ = .23, 90% CI = [.17, .24]. The effect of group was not significant, *F* < 1, and there were no other significant interactions of factors, *F* < 1. Post hoc *t* tests (Holm–Bonferroni corrected) that explored the effect of cue showed there was no significant difference between cues A, B, and X (*p* > .5), but each of those cues elicited significantly greater responding than the nonreinforced Cue Y (*p* < .001). Simple main effects analysis of the cue by session interaction revealed that there was a significant effect of cue from Session 3 onward (*p* < .04), but not before (*p* > .20).[Fig fig1]

A Bayesian 2 (group) by 3 (cue: A, B, and Y) by 12 (session) ANOVA was conducted to test evidence for the null hypothesis that there was no difference in the acquisition of responding to partially and continuously reinforced cues. For the cue by group interaction, BF_incl_ = 0.37. For the cue by group by session interaction, BF_incl_ = 1 × 10^−5^. Therefore, the results provided at least 2.7 times evidence for the null hypothesis over the alternative hypothesis.

Additional analyses were conducted comparing response rates between cues under matched conditions (see the online supplemental materials). The analyses compared responding across similar durations within the first trial of each trial type across sessions in the latter half of training (see Supplemental Figure 3 in the online supplemental materials). There was no significant difference between partially and continuously reinforced cues, and Bayesian analyses found evidence in favor of the null hypothesis.

Pre-CS responding, collapsed across trial types, reduced over the course of training for both groups (see Figure 1). There was a significant effect of session, *F*(11, 330) = 26.19, *p* < .001, η_p_^2^ = .47, 90% CI = [.39, .50], no significant effect of group, *F*(1, 30) = 1.80, *p* = .18, and no significant interaction of group and session, *F*(11, 330) = 1.33, *p* = .21.

#### Stage 2

**Figure 2 fig2:**
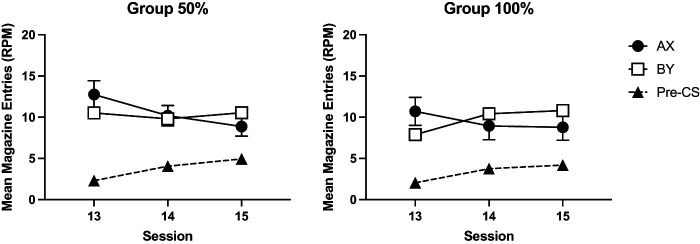
Compound Conditioning in Stage 2, Experiment 1 *Note.* Mice initially showed summation of conditioned responding by responding more to AX than BY. Mean magazine entries are shown as RPM. The error bars for AX data indicate ±*SEM* of the within-subjects difference between AX and BY. RPM = rate per minute; AX = compound AX; BY = compound BY; CS = conditioned stimulus; *SEM* = standard error of the mean.

Performance during the compound conditioning phase is shown in Figure 2. Mice initially showed greater responding to the compound AX than to BY but by the third session response rates for AX reduced to a level that was less than for BY. A 2 (compound: AX, BY) by 2 (group: 50%, 100%) by 3 (session) ANOVA revealed that there was no significant effect of compound, *F* < 1, but there was a significant compound by session interaction, *F*(2, 60) = 17.66, *p* < .001, η_p_^2^ = .37, 90% CI = [.20, .49]. Simple main effects analysis showed that there was a significant effect of session for AX, *F*(2, 60) = 11.59, *p* < .001, but not for BY, *F*(2, 60) = 2.5, *p* = .09. There was a significant effect of compound on Session 1, *F*(1, 30) = 4.44, *p* = .04, but not on Sessions 2 and 3, largest *F*(1, 30) = 3.59, *p* = .07. The effect of session was not significant, *F*(2, 60) = 1.12, *p* = .33. There were no other significant main effects or interactions of factors, *F* < 1.[Fig fig2]

Pre-CS response rates increased over the course of Stage 2 training. There was a significant effect of session, *F*(2, 60) = 11.08, *p* < .01, η_p_^2^ = .27, 90% CI = [.11, .39]. There was no significant effect of group or interaction between group and session, *F* < 1.

#### Test

**Figure 3 fig3:**
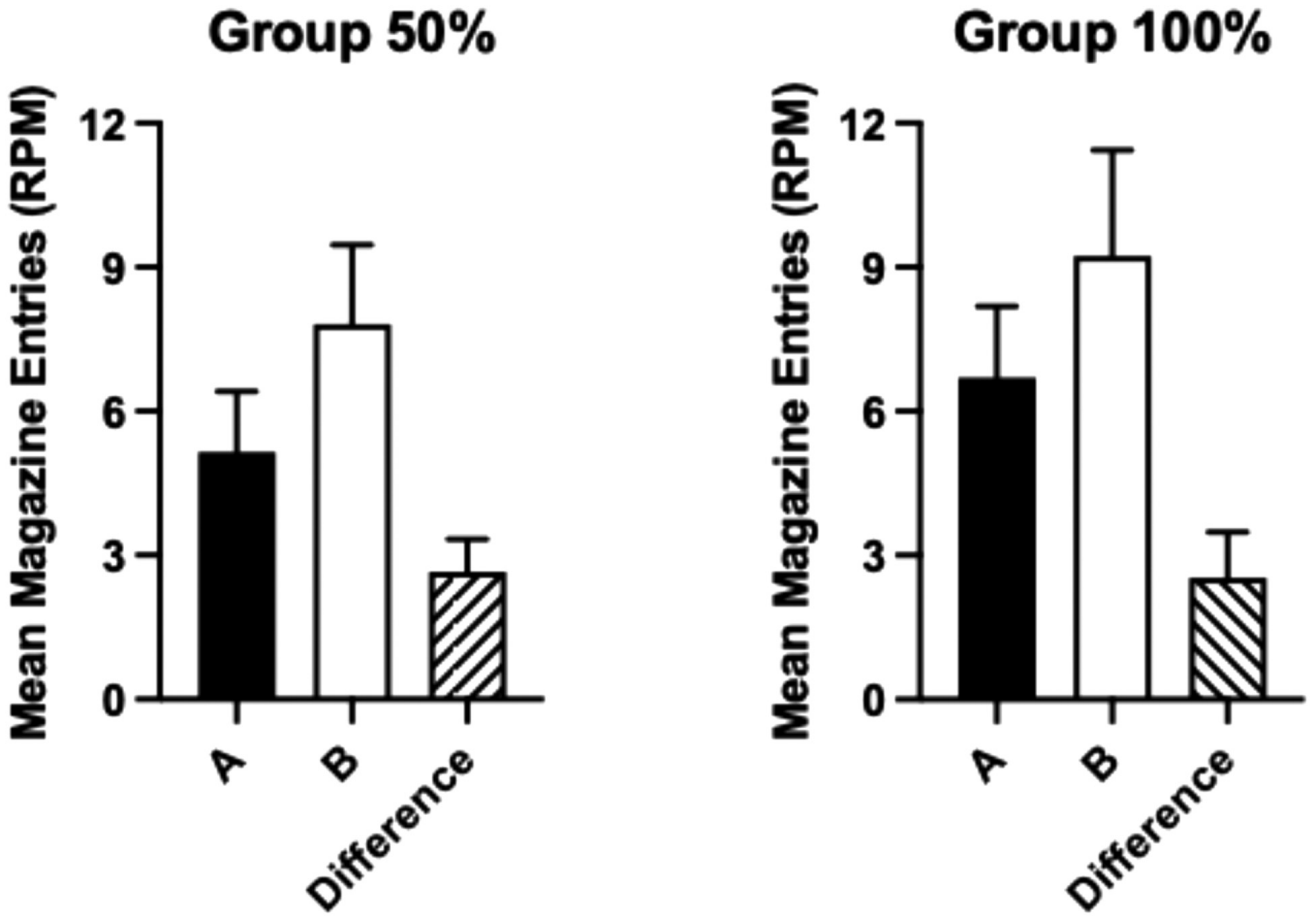
Overexpectation Test, Experiment 1 *Note.* Mice responded less to A, the overexpectation cue than B, the control cue. The striped bar indicates the mean difference in response rates between Cue B and A. Mean magazine entries are shown as RPM. Error bars indicate the standard error of the mean. RPM = rate per minute; A = Cue A; B = Cue B.

The results of the overexpectation test are shown in Figure 3. Both groups responded more to Cue B than Cue A and the extent of the difference in responding to cues was similar across groups. In a 2 (cue: A, B) × 2 (group) ANOVA, it was found that there was a significant effect of cue, *F*(1, 30) = 19.56, *p* < .001, η_p_^2^ = .39, 90% CI = [.16, .55]. The effect of group and the cue by group interaction was not significant, *F* < 1.[Fig fig3]

In order to test the strength of evidence for the null hypothesis, a Bayes factor was calculated in JASP using the default priors, testing the prediction that the overexpectation effect would be greater for Group 100% than Group 50%. A Bayesian between-subjects *t* test was conducted comparing the difference between response rates for the two cues (response rate for Cue B minus the response rate for A). BF_10_ = 0.31, thus providing 3.2 times evidence for the null hypothesis over the alternative hypothesis.

**Figure 4 fig4:**
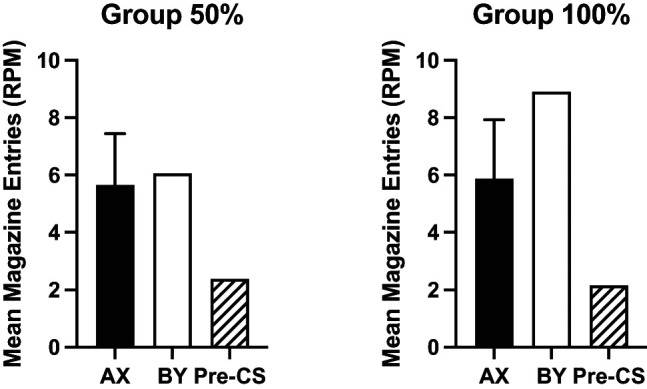
Responding to the Reinforced Compounds During the Overexpectation Test, Experiment 1 *Note.* Mean magazine entries are shown as RPM. The error bars for AX indicate the standard error of the mean of the within-subjects difference between AX and BY. RPM = rate per minute; AX = compound AX; BY = compound BY; CS = conditioned stimulus.

An additional analysis of responding to the reinforced compounds, AX and BY, and the pre-CS rates of responding (see Figure 4) showed no significant effect of group, *F* < 1, or interaction of factors, *F* < 1. Response rates significantly differed between the compounds and the pre-CS period, *F*(2, 60) = 9.77, *p* < .001, η_p_^2^ = .25, 90% CI = [.09, .37]. Post hoc *t* tests (Holm–Bonferroni corrected) showed that there was no significant difference between responses to the two reinforced compounds (*p* = .65), but pre-CS response rates were significantly lower than both compounds, *p* < .003.[Fig fig4]

The probability of reinforcement per trial failed to affect overexpectation. Therefore, the results did not support the hypothesis that negative prediction error reflects an expectation of reinforcement that is dependent on probability information. As anticipated, there was also no significant effect on acquisition of responding. There was also no significant effect on summation of conditioned responding consistent with other findings showing that responding to compounds reflects the summed rates of reinforcement of the individual cues (Andrew & Harris, 2011; Harris et al., 2012).

## Experiments 2a and 2b

The primary purpose of Experiments 2a and 2b was to rule out potential accounts of the failure to observe an effect of probability of reinforcement per trial in Experiment 1. In Experiment 1, manipulation of probability was achieved by Group 50% switching from 50% reinforcement per trial in Stage 1 to 100% in Stage 2. It is possible that the reduced responding in Group 50% to Cue A compared to Cue B was because of generalization decrement caused by the manipulation of probability and/or the average trial duration between Stages in 1 and 2 rather than overexpectation. Such an effect may have masked an effect of probability of reinforcement per trial on overexpectation. Therefore, in Experiments 2a and 2b, the procedure of Experiment 1 was replicated but instead of Group 50% switching from partial reinforcement in Stage 1 to continuous reinforcement in Stage 2, the probability of reinforcement was now manipulated by Group 100% switching to 50% reinforcement in Stage 2 (see Table 2). If overexpectation is observed when Group 50% are maintained on 50% reinforcement per trial for both Stages 1 and 2, then this would rule out the possibility that the low responding to A compared to B, in Experiment 1, was a consequence of generalization decrement across the stages. Furthermore, if overexpectation is dependent on the probability of reinforcement per trial, then shifting Group 100% to 50% reinforcement in Stage 2 should result in a larger overexpectation effect than for Group 50%. On the basis that summation of learning reflects summation of expected amount of reinforcement and with the assumption that probability of reinforcement linearly relates to associative strength, while Group 50% should expect one reinforcement per trial in Stage 2 rather than half on average, Group 100% should expect two lots of reinforcement rather than half.[Table tbl2]

Another motivation for Experiments 2a and 2b was to test the effect of probability of reinforcement per trial on overexpectation after fewer compound conditioning trials. It is possible that probability of reinforcement per trial has an effect on initial decrements in responding in overexpectation, but the effect diminishes across compound conditioning. Such an effect has been found in extinction learning in which the probability of reinforcement per trial affects the number of nonreinforced trials required before a decrement in responding is observed (Chan & Harris, 2017, 2019; Harris & Andrew, 2017). Thus, cues associated with a low probability of reinforcement require more nonreinforced trials before extinction is observed than cues with a higher probability of reinforcement. Because the minimum number of trials necessary for overexpectation to occur was not known, we chose to present 24 trials of AX and BY each reinforced on 50% of trials before presenting probe trials of Cues A and B intermixed with additional partially reinforced compound trials. The 12 reinforced compound trials, before the first probe trial, in which overexpectation may occur, were fewer than in Experiment 1 in which mice received 27 reinforced compound trials of each trial type before the first probe trial. The results show that 12 reinforced trials were sufficient for a reduction in summation to be observed over sessions.

Experiment 2b also allowed the inclusion of an additional control procedure for overexpectation. In Experiment 1, Cue Y was presented in compound with Cue B in Stage 2. Cue Y had been nonreinforced in Stage 1 and mice had learnt the discrimination between Cue Y and reinforced Cues A, B, and X. Although Cue Y had lower associative strength than the reinforced cues, it is possible that it may have undergone other processes that may have affected its ability to function as a control for Cue X in as far as being associatively neutral. It is possible that during Stage 1 it may have come to predict the absence of reinforcement such that it acquired inhibitory associative strength. Indeed, if the context gained excitatory associative strength, then Cue Y would have undergone feature-negative training, which, typically, establishes a cue as conditioned inhibitor (Rescorla, 1969). Although we have found no evidence of this in similar discrimination training procedures (Austen et al., 2022), if this was the case, then subsequent reinforcement of the BY compound may have led to further increments in the associative strength of B because the combined associative strength of the compound BY was lower than for B alone. In other words, the inhibitory associative strength of Cue Y allows B to undergo superconditioning (Williams & McDevitt, 2002). This would result in Cue B gaining greater associative strength than Cue A such that the difference between responding to the cues in the test phase would not reflect overexpectation alone. In order to avoid the possibility of Cue Y being inhibitory in Stage 2, in Experiment 2b, Cue Y was a novel cue at the start of Stage 2. During Stage 1, instead of nonreinforced exposure to Cue Y, mice received nonreinforced trials of Cue Z, which was a different cue but from the same modality as Cues X and Y.

A final purpose of Experiments 2a and 2b was to examine posttrial response rates. While a number of studies have demonstrated that acquisition of conditioned responding is sensitive to reinforcement rate and not probability of reinforcement per trial (Chan & Harris, 2017, 2019; Harris, 2019; Harris & Andrew, 2017; Harris et al., 2015), it has been found that responding in the period immediately after the CS offset reflects the probability of reinforcement (Harris & Kwok, 2018). Thus, when two cues differed in probability of reinforcement per trial but were matched for overall reinforcement rate, the rats showed greater responding during the post-CS period for the cue with a high probability of reinforcement per trial compared to the other CS despite responding during the two CSs being similar. A reason why examining post-CS response rates would be useful given the results of Experiment 1 is that it may allow detection of an effect of probability of reinforcement per trial in the absence of an effect on overexpectation and summation during CS responding. Because both groups received partial reinforcement during Stage 2 compound training, it was possible to compare post-CS response on the nonreinforced trials for compounds AX and BY (see Table 2) across both groups.

### Method

#### Experiment 2a

##### Subjects

Thirty-two naïve female C57BL/6J mice (Charles River UK Ltd), approximately 10 weeks old at the start of testing, with a mean free-feeding weight of 17.8 g (range = 15.5–19.6) were used. All other details were the same as Experiment 1.

##### Apparatus

All other details were the same as Experiment 1.

##### Procedure

Mice were randomly assigned to one of two groups (*N* = 16 per group). In Stage 1, Group 50% received 12 sessions of training with four cues, A, X, B, and Y that were, on average across trials, 10 s in duration. Cues A, B, and X were reinforced on a random 50% of trials. Cue Y was nonreinforced. Group 100% received similar training except that Cues A and X were variable duration 20 s cues rather than 10 s and were reinforced on 100% of trials. Trial types were presented in a random order with the following constraints. For Group 50%, each block of eight trials consisted of two trials of each trial type (A, B, X, and Y). For Group 100%, each block of six trials consisted of one trial of A and X each and two of B and Y each. Within each session, Group 100% received three trials of A and X each and six of B and Y each. Group 50% received six trials of each trial type. The difference in trial numbers for the two groups resulted in both groups receiving the same cumulative exposure to Cues A and X and the same number of pairings with reinforcement. In order to equate the session lengths, the fixed intertrial interval for Group 50% was 90 s (from CS offset to CS onset), and for Group 100%, it was 120 s. The sessions lasted 40 min. The allocation of stimuli to trial types across mice was counterbalanced in the same manner as used for Experiment 1.

On Sessions 13, 14, 17, and 18, mice received 12 compound presentations of AX and BY, and each was reinforced on a random 50% of trials. Compound presentations were a variable duration of 10 s. The order of trial types was random with the constraint that were equal numbers of each trial type every four trials. The intertrial interval was a fixed duration of 90 s. The sessions lasted 40 min.

On sessions 15 and 16 and 19–21, mice received 50% reinforced presentations of compounds AX and BY and also nonreinforced presentations of A and B. Compounds AX and BY were presented for a variable duration 10 s, but Cues A and B were presented for a fixed duration of 10 s. On Sessions 15 and 16, there were two trials of A and B each and 10 presentations of AX and BY each. On Session 15, Trials 1 and 2 consisted of one trial each of A and B. This was also true for Trials 23 and 24. On Session 16, Trials 13 and 14 consisted of one trial each of A and B. This was also true for Trials 23 and 24. On Sessions 19 and 20, there were four trials of A and B each and eight trials of AX and BY each. For both Sessions 19 and 20, Trials 5 and 6, 11 and 12, 17 and 18, and 23 and 24 consisted of one trial each of A and B. On Session 21, there were eight trials of A and B each and four trials of AX and BY each. A and B trials occurred on Trials 5–12 and 17–24, in a double alternating manner. For Sessions 15, 16, and 19–21, the order of A and B across the trials was counterbalanced within groups with respect to the allocation of stimuli to trial types across mice. The order of AX and BY was random. The sessions lasted 40 min.

#### Experiment 2b

##### Subjects and Apparatus

Thirty-two naïve female C57BL/6J mice (Charles River UK Ltd), approximately 10 weeks old at the start of testing, with a mean free-feeding weight of 18.3 g (range = 16.5–20.0) were used. All other details were the same as Experiments 1 and 2a. The apparatus was the same as Experiments 1 and 2a except an additional cue was provide by a pure tone generator (ENV-323AM) that produced a 2,900 Hz tone at 80 dB.

##### Procedure

The procedure was similar to Experiment 2a but with a number of exceptions. The counterbalancing of the allocation of stimuli to Cues A, B, X, and Y was the same as Experiment 2a except that the alternating flashing left and right LED stimulus was replaced with a flashing presentation of the right LED alone. In Stage 1, in contrast to Experiment 2a, mice did not received trials of Y but instead received nonreinforced presentations of Cue Z. For mice that were allocated an auditory stimulus for Cue X (noise/clicker), Cue Z was the pure tone. For mice that were allocated a visual stimulus for Cue X (house light/flashing right LED), Cue Z was a constant presentation of the left LED. The replacement of Cue Y with Cue Z in Stage 1 resulted in Cue Y being novel at the start of Stage 2 training.

##### Statistical Analyses

The results of both Experiments 2a and 2b were combined and analyzed with experiment included as a factor. All other details were the same as Experiment 1.

### Results and Discussion

#### Stage 1: Acquisition

**Figure 5 fig5:**
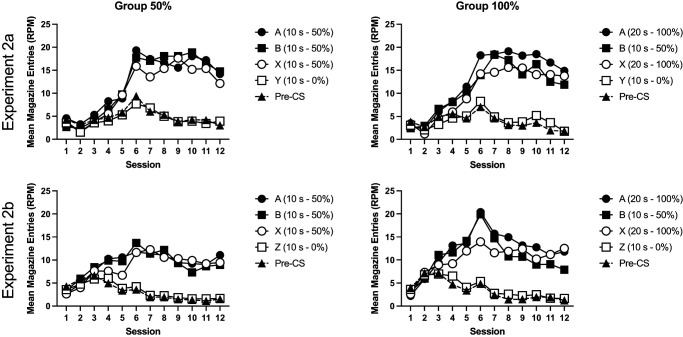
Acquisition of Conditioned Responding in Stage 1, Experiments 2a and 2b *Note.* Mean magazine entries are shown as RPM. Cues A, B, and X were reinforced. In Experiment 2a, Cue Y was nonreinforced. In Experiment 2b, Cue Z was nonreinforced. For each cue, the durations and percentages indicate the mean trial duration and probability of reinforcement per trial. RPM = rate per minute; A = Cue A; B = Cue B; X = Cue X; Y = Cue Y; CS = conditioned stimulus.

Acquisition of responding is shown in Figure 5. Mice acquired conditioned responding to Cues A, B, and X over the course of training. Responding to the nonreinforced cue (Y in Experiment 2a and Z in Experiment 2b) initially increased at the start of training but then subsequently decreased. A 4 (cue: A, B, X, and Y) by 12 (session) by 2 (group: 50%, 100%) by 2 (experiment) ANOVA showed a significant effect of cue, *F*(3, 180) = 29.19, *p* < .001, η_p_^2^ = .24, 90% CI = [.15, .32], session, *F*(11, 660) = 52.92, *p* < .001, η_p_^2^ = .47, 90% CI = [.42, .50], and cue by session interaction, *F*(33, 1980) = 16.17, *p* < .001, η_p_^2^ = .21, 90% CI = [.17, .22]. Post hoc tests (Holm–Bonferroni corrected) showed that mice responded more to Cues A, B, and X compared to the nonreinforced cue (Y/Z), *p* < .001. There were no significant differences in responding to the reinforced cues (smallest noncorrected *p* = .036, corrected α = .0167). Simple main effects analysis of the interaction showed that there was no significant effect of cue for the first three sessions, *F* < 1, but there was for the remaining sessions, smallest *F*(3, 58) = 4.98, *p* = .004. There was no significant effect of group, *F* < 1, or experiment, *F*(1, 60) = 3.09, *p* = .08. There was a significant session by experiment interaction, *F*(11, 660) = 15.26, *p* < .001, η_p_^2^ = .20, 90% CI = [.15, .23]. The three-way cue by session by experiment interaction failed to reach significance, *F*(33, 660) = 1.96, *p* = .051. No other interactions of factors were significant, *p* > .2. Simple main effects analysis of the session by experiment interaction showed that mice from Experiment 2a responded at a significantly lower level than Experiment 2b in Sessions 2–4 but at a significantly higher level than Experiment 2b on Sessions 7–12, smallest *F*(1, 60) = 6.14, *p* = .02. There was no significant difference between experiments on the remaining sessions, *p* > .2.[Fig fig5]

A Bayesian ANOVA was conducted to test evidence for the null hypothesis that there was no difference in the acquisition of responding to partially and continuously reinforced cues. For the cue by group interaction, BF_incl_ = 0.019. For the cue by group by session interaction, BF_incl_ = 2 × 10^−15^. Therefore, the results provided at least 52 times greater evidence for the null hypothesis over the alternative hypothesis.

Additional analyses were conducted comparing response rates between cues under matched conditions (see the online supplemental materials). The analyses compared responding across similar durations within the first trial of each trial type across sessions in the latter half of training (see Supplemental Figure 4 in the online supplemental materials). There was no significant difference between partially and continuously reinforced cues and Bayesian analyses found evidence in favor of the null hypothesis.

Pre-CS response rates initially increased and then declined over the course of training. A 2 (group) by 2 (experiment) by session ANOVA revealed a significant effect of session, *F*(11, 660) = 31.35, *p* < .001, η_p_^2^ = .34, 90% CI = [.29, .38], and experiment, *F*(1, 60) = 10.20, *p* < .001, η_p_^2^ = .14, 90% CI = [.03, .28], but no significant effect of group, *F* < 1. There was a significant experiment by session interaction, *F*(11, 660) = 19.25, *p* < .001, η_p_^2^ = .33, 90% CI = [.19, .28], reflecting that mice in Experiment 2a responded at a lower rate than mice in Experiment 2b early on in training but came to respond more than Experiment 2b later on in training. Simple main effects analysis showed a significant effect of experiment on Sessions 2–3 and 5–12, smallest *F*(1, 60) = 7.17, *p* = .01. There was no significant effect on Sessions 1 and 4, *F* < 1. There were no other significant interactions of factors, *p* > .06.

#### Stage 2

**Figure 6 fig6:**
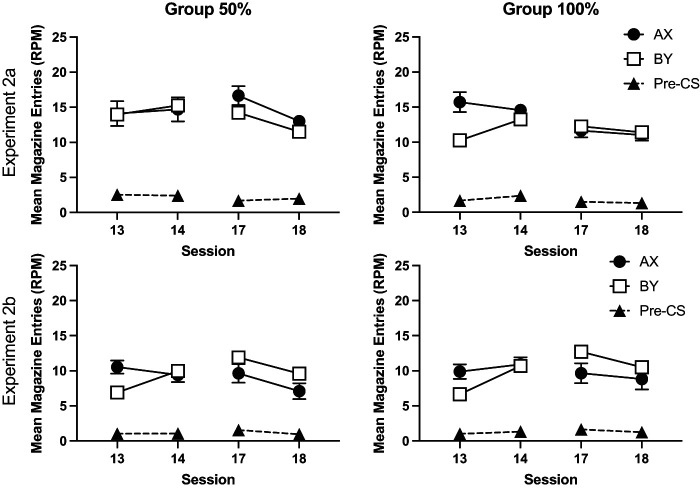
Compound Conditioning in Stage 2, Experiments 2a and 2b *Note.* Mice initially showed summation of conditioned responding by responding more to AX than BY. Mean magazine entries are shown as RPM. The error bars for AX data indicate ±*SEM* of the within-subjects difference between AX and BY. RPM = rate per minute; AX = compound AX; BY = compound BY; CS = conditioned stimulus; *SEM* = standard error of the mean.

Performance during the compound training phase is shown in Figure 6. In Session 13, the first session of compound training, mice responded more to AX than BY. This effect was not evident in Session 14. The results of the first block of compound training (Sessions 13 and 14) were analyzed using a 2 (compound: AX, BY) by 2 (session) by 2 (group: 50%, 10%) by 2 (experiment) ANOVA. There were significant effects of compound, *F*(1, 60) = 7.91, *p* < .007, η_p_^2^ = .12, 90% CI = [.02, .25], session, *F*(1, 60) = 7.60, *p* < .008, η_p_^2^ = .11, 90% CI = [.02, .24], and a significant compound by session interaction, *F*(1, 60) = 30.57, *p* < .001, η_p_^2^ = .34, 90% CI = [.18, .47]. Simple main effects analysis of the interaction revealed that mice responded more to AX than BY on Session 13, *F*(1, 60) = 21.74, *p* < .001, but not on the Session 14, *F* < 1. Responding to BY significant increased over sessions, *F*(1, 60) = 27.29, *p* < .001, but responding to AX did not significantly differ across sessions, *F* < 1. There was a significant effect of experiment, *F*(1, 60) = 7.05, *p* = .01, η_p_^2^ = .11, 90% CI = [.01, .23]. The effect of group was not significant, *F* < 1. There were no other significant interactions of factors, *p* > .10, except for a significant four-way interaction, *F*(1, 60) = 4.57, *p* = .04, η_p_^2^ = .07, 90% CI = [.002, .19]. Inspection of the results for each experiment suggested that the summation effect on the first session was weaker in the 50%–50% group compared to 100%–50% in Experiment 2a. Separate analyses of each experiment revealed that for both experiments, the three-way interaction was not significant, although approaching significance in Experiment 2a, *F*(1, 30) = 4.10, *p* = .052, but not Experiment 2b, *F* < 1.[Fig fig6]

**Figure 7 fig7:**
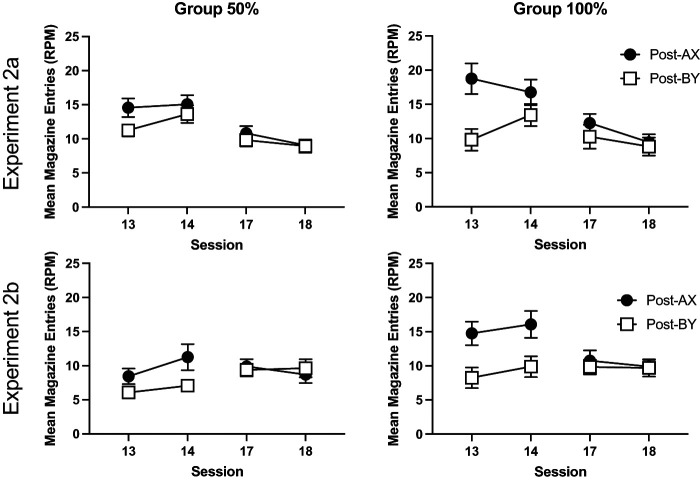
Post-CS Responding in the Compound Conditioning Stage of Experiments 2a and 2b *Note.* Group 100% initially showed greater responding to AX than Group 50%. Mean magazine entries are shown as RPM. Note that in contrast to previous figures, the error bars for post-AX and post-BY indicate ±*SEM*. CS = conditioned stimulus; RPM = rate per minute; AX = compound AX; BY = compound BY; *SEM* = standard error of the mean.

Responding during the 10 s period immediately after the presentation of the compounds on nonreinforced trials is shown in Figure 7. In the first session of compound training, Session 13, mice responded more in the period immediately after AX than the period after BY, and this effect was greater for Group 100% than Group 50%. These effects were less apparent in the subsequent sessions. The data from the first block of training, Sessions 13 and 14, were analyzed using a 2 (compound: AX, BY) by 2 (session) by 2 (group: 50%, 100%) by 2 (experiment) ANOVA. There was a significant effect of compound, *F*(1, 60) = 71.69, *p* < .001, η_p_^2^ = .54, 90% CI = [.40, .64], that significantly interacted with group, *F*(1, 60) = 10.13, *p* < .002, η_p_^2^ = .14, 90% CI = [.03, .28], and there was also a significant cue by group by session interaction, *F*(1, 60) = 4.31, *p* = .042, η_p_^2^ = .12, 90% CI = [.07, .18]. Simple main effects analysis of the cue by group interaction revealed that Group 100% responded significantly more than Group 50% in the post-CS period for AX, *F*(1, 60) = 7.14, *p* = .01, but there was no significant difference between groups in the post-CS period for BY, *F* < 1. Both groups responded more in the post-CS period for AX than BY, smallest, *F*(1, 60) = 13.96, *p* < .001. There was a significant effect of session, *F*(1, 60) = 10.07, *p* < .002, η_p_^2^ = .14, 90% CI = [.03, .28], a cue by session interaction, *F*(1, 60) = 4.50, *p* = .04, η_p_^2^ = .07, 90% CI = [.002, .19], and a three-way cue by session by experiment interaction, *F*(1, 60) = 10.12, *p* = .002, η_p_^2^ = .14, 90% CI = [.03, .28]. Separate analyses of each experiment revealed a significant cue by session experiment for Experiment 2a, *F*(1, 60) = 13.74, *p* < .001, but not Experiment 2b, *F* < 1. There were no other significant interactions of factors, *F* < 1.[Fig fig7]

The analysis of pre-CS rates of responding revealed that mice in Experiment 2a responded more than mice in Experiment 2b, *F*(1, 60) = 17.05, *p* < .001, η_p_^2^ = .22, 90% CI = [.08, .36]. There were no other significant effects of group, experiment or session, or interactions of factors (*p* > .06).

The second block of compound training, Sessions 17 and 18, after the initial test sessions, was analyzed separately, in a similar manner as the first block. During the presentation of the compounds, responding significantly decreased over the two sessions, *F*(1, 60) = 19.59, *p* < .001, η_p_^2^ = .25, 90% CI = [.10, .38]. There was a significant cue by experiment interaction, *F*(1, 60) = 8.49, *p* = .005, η_p_^2^ = .12, 90% CI = [.02, .25]. Simple main effects analysis revealed that this was because of significantly lower responding to AX than BY in Experiment 2b, *F*(1, 60) = 9.87, *p* = .003, but not Experiment 2a, *F* < 1. There were no other significant main effects or interactions, *p* > .06.

In the post-CS periods, responding reduced over the two sessions, *F*(1, 60) = 7.60, *p* = .008, η_p_^2^ = .11, 90% CI = [.02, .24]. There were no other significant main effects or interactions, *p* > .08. Pre-CS levels of responding were similar across groups. There were no significant effects or interactions of factors, *p* > .09.

#### Test

**Figure 8 fig8:**
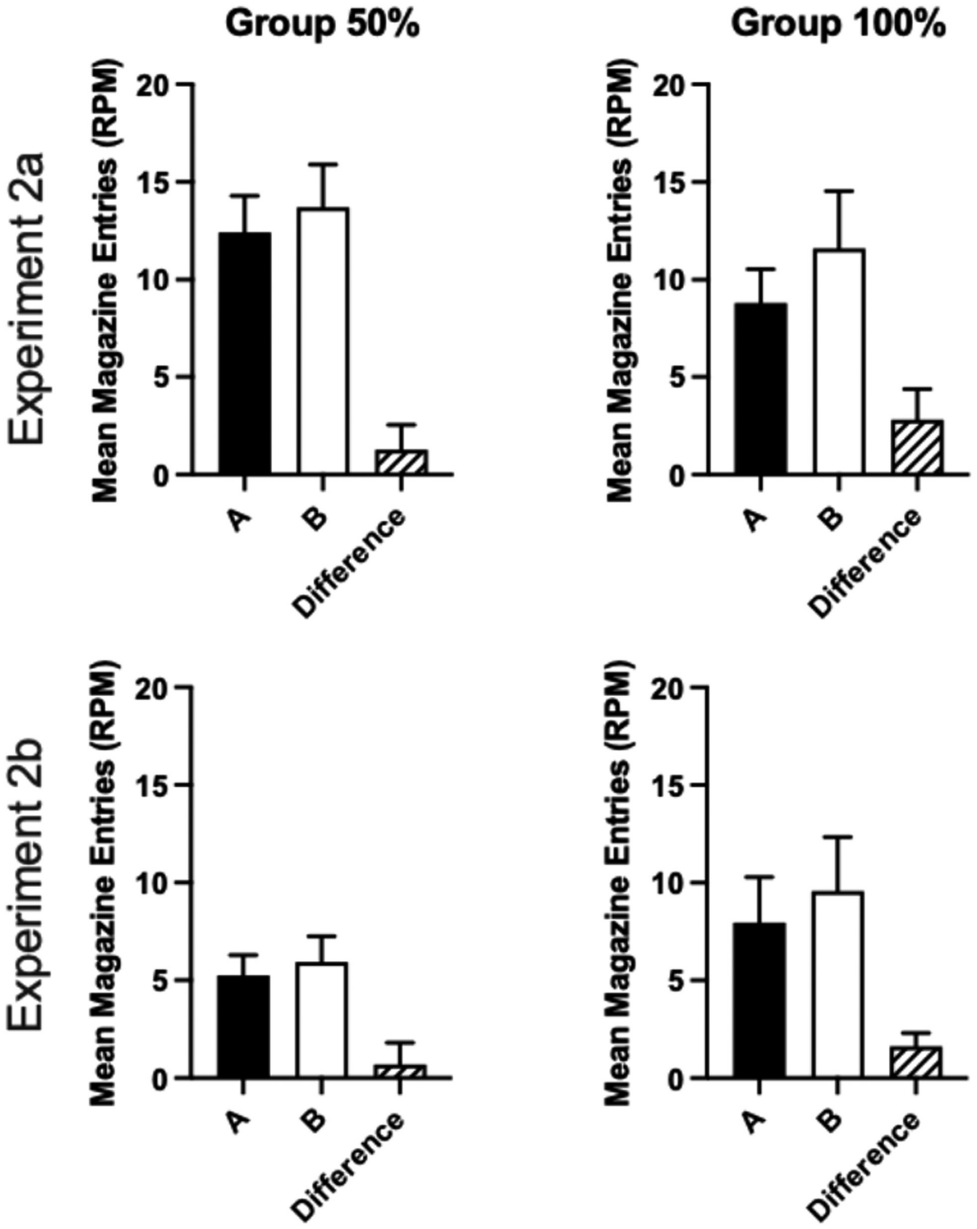
Overexpectation Test, Experiments 2a and 2b *Note.* Mice responded less to A, the overexpectation cue than B, the control cue. The striped bar indicates the mean difference in response rates between Cue B and A. Mean magazine entries are shown as RPM. Error bars indicate the standard error of the mean. RPM = rate per minute; A = Cue A; B = Cue B.

The response rates collapsed across test trials for each experiment are shown in Figure 8. Overall response rates were lower in Experiment 2b than 2a, but all groups showed lower rates of responding to A, the overexpectation cue, than the control cue B (see Supplemental Figure 1 in the online supplemental materials for the results broken down by trial). Mice responded more to cue B than cue A. A 2 (group: 50%, 100%) by 2 (cue: A, B) by 2 (experiment) ANOVA showed a significant effect of cue, *F*(1, 60) = 7.16, *p* = .01, η_p_^2^ = .11, 90% CI = [.02, .23]. The cue by group interaction was not significant, *F*(1, 60) = 1.07, *p* = .31. Mice in Experiment 2a responded at a significantly higher rate than mice in Experiment 2b, *F*(1, 60) = 4.79, *p* = .03, η_p_^2^ = .07, 90% CI = [.003, .19]. There were no other significant main effects or interactions of factors (smallest *p* = .14).[Fig fig8]

Although the cue by group interaction was not significant, the overexpectation effect was numerically smaller in Group 50% than Group 100%. In order to test the strength of evidence for the null hypothesis, a Bayes factor was calculated testing the prediction that the overexpectation effect would be greater for Group 100% than Group 50%. A Bayesian between-subjects *t* test was conducted comparing the difference between response rates for the two cues (response rate for cue B minus the response rate for A). BF_10_ = 0.67, thus providing 1.5 times evidence for the null hypothesis over the alternative hypothesis.

**Figure 9 fig9:**
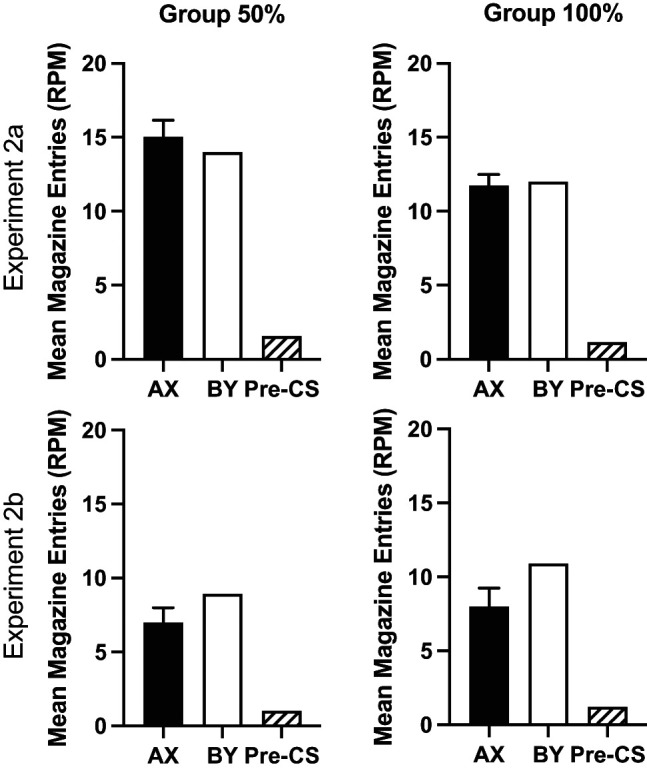
Responding to the Reinforced Compounds During the Overexpectation Test, Experiments 2a and 2b *Note.* Mean magazine entries are shown as RPM. The error bars for AX indicate the standard error of the mean of the within-subjects difference between AX and BY. RPM = rate per minute; AX = compound AX; BY = compound BY; CS = conditioned stimulus.

Responding during the reinforced compounds and the pre-CS periods (collapsed across all trial types) is shown in Figure 9. Response rates were analyzed using a 3 (period: pre-CS, AX, and BY) by 2 (experiment) by 2 (group: 50%, 100%) ANOVA. There was a significant effect of period, *F*(2, 120)= 81.17, *p* < .001, η_p_^2^ = .58, 90% CI = [.47, .64], a significant effect of experiment, *F*(1, 60) = 5.48, *p* = .023, η_p_^2^ = .08, 90% CI = [.006, .21], and significant period by experiment interaction, *F*(2, 120) = 5.04, *p* = .02, η_p_^2^ = .08, 90% CI = [.01, .15]. There were no other significant main effects or interactions, *F* < 1. Post hoc analysis of the effect of period confirmed that mice responded significantly more during AX and BY compared to the pre-CS period, *p* < .001, but there was no significant difference between AX and BY, *p* = .14. Simple main effects analysis of the experiment by period interaction revealed that mice in Experiment 2b responded at a significantly lower level than mice in Experiment 2a for AX, *F*(1, 30) = 9.62, *p* = .003, but not the pre-CS period or during BY, *p* > .1.[Fig fig9]

Similar to the results of Experiment 1, the probability of reinforcement failed to affect the strength of overexpectation. Therefore, regardless of whether Group 50% switched to 100% reinforcement for Stage 2 compound training, as in Experiment 1, or Group 100% switched to 50% compound training, as in Experiments 2a and 2b, the manipulation of probability of reinforcement per trial failed to affect the extent of overexpectation. Furthermore, the extent of overexpectation was similar across the experiments, suggesting that whether Cue Y was novel or not made little difference to the pattern of results.

Although there was no significant cue by group interaction, the overexpectation effect was numerically smaller in the 50% groups compared to the 100% groups. Bayesian analysis, however, found evidence in favor of the null hypothesis that there was no difference between groups. While the cue by group by experiment three-way interaction was not significant, it is possible that the ability to detect a cue by group interaction depended on the different treatments in Experiments 2a and 2b. Separate analyses of each experiment (see the online supplemental materials) failed to reveal evidence of a cue by group interaction for either experiment (*F* < 1). It is possible that the study was not sensitive enough to detect a cue by group interaction; however, there was no evidence for a cue by group interaction from the statistical analysis and, furthermore, the lack of effect of group in Experiment 2 is consistent with the lack of effect in Experiment 1.

One concern with Experiment 1 was that if probability of reinforcement per trial affects the rate at which overexpectation occurs but not the overall extent of overexpectation then overtraining in the compound training phase may have masked a possible effect. This is unlikely to be the case in Experiment 2 because, although variable over trials, there was a trend, albeit nonsignificant, for the overexpectation effect to emerge over training trials (see Supplemental Figure 1 in the online supplemental materials). Therefore, the test of overexpectation likely occurred at the earliest measurable point.

The lack of effect of probability of reinforcement per trial on overexpectation is in contrast to the effects observed in post-CS responding. During the summation test, the probability of reinforcement failed to affect summation during the CS, but in the post-CS periods, Group 100% responded more to AX than Group 50%. Therefore, the post-CS summation effect was greater for Group 100% than Group 50% despite no significant difference in the extent of summation during presentation of the compound. Rather than being an effect on summation per se, this likely reflects that for Group 100%, Cues A and X individually signaled 100% probability of reinforcement, whereas for Group 50%, the cues signaled 50% reinforcement.

Experiment 2b was conducted because of the possibility that the effect in Experiment 1, rather than reflecting overexpectation, may instead be the result of increased associative strength of Cue B rather than a reduction in associative strength of Cue A. This could have occurred because, during Stage 1, Cue Y may have acquired inhibitory associative strength through signaling a reduction in the occurrence of the US compared to the context (Miller et al., 1991). In Stage 2, when the compound BY was reinforced, the inhibitory associative strength of Cue Y would have led to positive prediction error that would allow Cue B to increase in associative strength. To rule out this possibility, Cue Y was a novel cue in Experiment 2b and, therefore, was associatively neutral. It was found that the extent of overexpectation, as indicated by the difference in responding between Cues A and B, did not differ between Experiment 2b and 2a in which Cue Y was a nonreinforced cue during Stage 1. It is possible, however, that a novel cue may result in external inhibition such that there is an increase in the prediction error at the start of training on reinforced trials with BY compared to the previous training with B alone. For this to result in greater responding to Cue B than to Cue A in the test phase, it would have to be assumed that the effect of external inhibition is greater for Cue Y than Cue X. But if external inhibition is caused by generalization decrement that results in a failure of learning to transfer completely between training with the Element B and the Compound BY then the potential for external inhibition would be matched between the Cues Y and X. Therefore, this account would not explain a difference in responding between Cues A and B. Furthermore, even if Cue Y was able to elicit greater external inhibition than Cue X, then any new learning about Cue B as a consequence of compound BY training would have to generalize to B when presented alone. This may not happen if generalization decrement occurs as a consequence of differing representations of cues during elemental and compound training (Brandon et al., 2000; Pearce, 2002). Nonetheless, it is possible that Cue Y, as a novel cue was more salient than the pretrained Cue X and, therefore, had greater potential for external inhibition. It has been found, however, that novel cues are more effective at restricting excitatory learning of other cues than familiar cues that have had nonreinforced preexposure (Navarro et al., 1989). Because there was no significant difference in the overexpectation effect between Experiments 2a and 2b, it seems unlikely that Cue Y as either a novel cue (Experiment 2b) or a preexposed nonreinforced cue (Experiment 2a) resulted in increased learning of B.

While the results of Experiment 2 are consistent with Experiment 1 and other demonstrations of an overexpectation effect, it is possible that it not a pure test of overexpectation. Experiment 2 differs from Experiment 1 in that in Stage 2, compound training, the compound was partially reinforced rather than continuously reinforced. If extinction is a trial-dependent process, then on nonreinforced trials, the amount of extinction that occurs for AX would be expected to be greater than for BY. This is because the discrepancy between the expectation of the US and nonreinforcement on AX compound trials is greater than for BY. According to error-correction models of learning (e.g., Rescorla & Wagner, 1972), this leads to a greater reduction in associative strength. Therefore, AX may have undergone greater extinction on nonreinforced trials than BY in addition to the reduction caused by overexpectation on reinforced trials. This depends on extinction occurring in a trial-by-trial manner rather than continuously over cumulative nonreinforced exposure. The fact that Experiment 1, in which compounds AX and BY were continuously reinforced, produced similar results to Experiment 2 results suggests that the potential for deepened extinction did not qualitatively change the pattern of results in Experiment 2. Thus, the manipulation of probability of reinforcement per trial did not affect reductions in conditioned responding as a consequence of overexpectation alone in Experiment 1 or overexpectation with the potential for extinction in Experiment 2.

## Experiment 3

The lack of effect of probability of reinforcement per trial on overexpectation in Experiments 1 and 2 is in contrast its effect on extinction of magazine approach behavior in rats (Chan & Harris, 2019). While this may suggest a dissociation between overexpectation and extinction, there are numerous differences between the procedures used in Experiments 1 and 2, which failed to find an effect, and those used for extinction learning other than the difference in species. The purpose of Experiment 3 was to test the effect of probability of reinforcement per trial on extinction learning using procedures similar to those used in Experiments 1 and 2 (see Table 3). Thus, the manipulation of probability of reinforcement per trial was between subjects: one group was trained with a 50% reinforced cue that was on average 10 s in duration (Group 50%). The other group was trained with a 100% reinforced cue that was on average 20 s in duration (Group 100%). The manipulation of extinction was within subjects: mice were initially trained with two cues (A and B) before one cue was nonreinforced in the extinction phase (Cue B) and other continued to be reinforced (Cue A). In order to control for any differences in generalization between cues on the basis of cue duration and probability of reinforcement per trial, for half of the mice within each group, the reinforced cue was a variable duration 10 s cue that was reinforced on 50% of trials (see Table 3). For the remaining mice within each group, the reinforced cue was a variable duration 20 s cue that was reinforced on 100% of trials. Therefore, within each group, the similarity between Cues A and B was balanced in terms of their trial durations and reinforcement contingencies.[Table tbl3]

### Method

#### Subjects and Apparatus

Thirty-two naïve female C57BL/6J mice (Charles River UK Ltd), approximately 9 weeks old at the start of testing, with a mean free-feeding weight of 18.3 g (range = 16.2–20.8) were used. One mouse died before the start of testing. All other details were the same as Experiment 1 except that only the auditory cues (noise and clicker) were used.

#### Procedure

Mice were randomly assigned to Group 100% (*N* = 15) or Group 50%. (*N* = 16). In Stage 1, mice were trained with two cues, A and B. Cue A was a cue that had a constant reinforcement contingency throughout Stage 1, the acquisition phase, and Stage 2, the extinction phase. Cue B was the cue that received nonreinforced extinction training in Stage 2. Within groups 100% and 50%, for half of the mice, Cue A was on average, across trials, 20 s in duration and reinforced on 100% of trials. For the remaining mice, Cue A was on average 10 s in duration and reinforced on a random 50% of trials. This counterbalancing resulted in four subgroups (see Table 3). In Stage 1, for Group 100%, Cue B was on average 20 s in duration and reinforced on 100% of trials. For Group 50%, Cue B was on average 10 s in duration and reinforced on a random 50% of trials. Cues that were 100% reinforced were presented 6 times per session. Cues that were 50% reinforced were presented 12 times per session. Thus, the number of CS–US pairings was matched between cues and groups. The intertrial interval differed between groups and subgroups in order that the overall session length was matched across groups. For mice trained with two 100% reinforced cues (A and B), the intertrial interval was 240 s. For mice trained with one 50% reinforced cue and one 100% reinforced cue, the intertrial interval was 160 s. For mice trained with two 50% reinforced cues, the intertrial interval was 120 s. The sessions lasted 52 min. There were six sessions of Stage 1. This resulted in the same number of CS–US pairing during acquisition as in Experiments 1 and 2. The allocation of stimuli (noise and clicker) to Cues A and B was counterbalanced within groups.

In Stage 2, for both groups, Cue B was on average 10 s in duration and now nonreinforced. Mice received 12 trials with Cue B per session. Cue A was presented and reinforced in the same manner as in Stage 1. For mice for which Cue A was 50% reinforced, the intertrial interval was 120 s. For mice for which Cue A was 100% reinforced, the intertrial interval was 160 s. The sessions lasted 52 min. Stage 2 lasted for six sessions.

#### Data and Statistical Analyses

The reinforcement contingencies of Cue A, either variable 10 s cue reinforced on 50% of trials or a variable 20 s cue reinforced on 100% of trials, did not significantly affect the outcome of the key factors of interest. Therefore, the results are presented collapsed across the factor of Cue A, and the factor is not included in the statistical analyses. The data (see Supplemental Figure 2 in the online supplemental materials) and additional analyses that include the factor Cue of A are presented in the online supplemental materials.

### Results and Discussion

#### Acquisition

**Figure 10 fig10:**
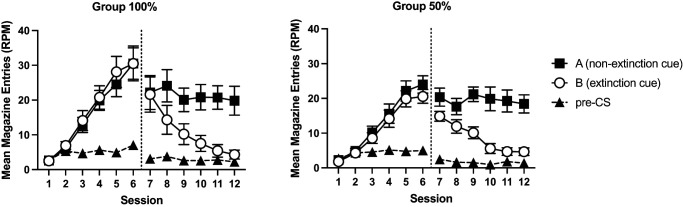
Stage 1 Acquisition (Sessions 1–6) and Stage 2 Extinction Training (Sessions 7–12) in Experiment 3 *Note.* Mice acquired responding to Cues A and B during Stage 1 and selectively extinguished responding to Cue B during Stage 2. Note that, similar to Figure 7, error bars indicate ±*SEM*. RPM = rate per minute; A = Cue A; B = Cue B; CS = conditioned stimulus; *SEM* = standard error of the mean.

In Stage 1, conditioned responding to Cues A and B increased similarly over sessions between the two groups (see Figure 10). A 2 (cue: A, B) × 6 (session) × 2 (group) ANOVA showed a significant effect of session, *F*(5, 145) = 63.38, *p* < .001, η_p_^2^ = .69, 90% CI = [.61, .73]. There was no significant effect of cue, *F* < 1. No other main effects or interactions were significant, *p* > .1.[Fig fig10]

A Bayesian ANOVA was conducted to test evidence for the null hypothesis that there was no difference in the acquisition of responding to partially and continuously reinforced cues. For the cue by group interaction, BF_incl_ = 0.78. For the cue by group by session interaction, BF_incl_ = 0.14. Therefore, the results provided at least 1.28 times greater evidence for the null hypothesis over the alternative hypothesis.

Additional analyses were conducted comparing the response rates between cues under matched conditions (see the online supplemental materials). The analyses compared responding across similar durations within the first trial of each trial type in the first extinction session (see Supplemental Figure 5 in the online supplemental materials). There was no significant difference between partially and continuously reinforced cues and Bayesian analyses found evidence in favor of the null hypothesis.

Pre-CS response rates, collapsed across trial types (see Figure 10), initially increased with training, but then subsequently reduced. A 2 (group) by 12 (session) ANOVA showed a significant effect of session, *F*(5, 145) = 5.04, *p* < .001, η_p_^2^ = .15, 90% CI = [.05, .21], but no significant effect of group, or interaction between session and group, *F* < 1.

#### Extinction Stage

Both groups reduced responding to Cue B over the course of extinction training while maintaining responding to Cue A (see Figure 10). A 2 (cue: A, B) × 6 (session) × 2 (group) ANOVA showed a significant effect of cue, *F*(1, 29) = 74.44, *p* < .001, η_p_^2^ = .72, 90% CI = [.55, .80], session, *F*(5, 145) = 9.68, *p* < .001, η_p_^2^ = .25, 90% CI = [.13, .32], and a significant cue by session interaction, *F*(5, 145) = 16.45, *p* < .001, η_p_^2^ = .36, 90% CI = [.24, .43]. The effect of group and remaining interactions of factors were not significant, *p* > .16.

A Bayesian ANOVA was conducted in order to assess the strength of evidence for the null hypothesis compared to the alternative hypothesis. To simplify the analysis, the effect of cue (reinforced vs. nonreinforced) was reduced to the difference between response rates such that a group by session ANOVA was conducted. For the effect of group, BF_incl_ = 0.35 and for the group by session interaction, BF_incl_ = 0.46. Therefore, evidence for the null hypothesis was at least 2.18 times greater than for the alternative hypothesis.

Pre-CS response rates reduced over the extinction phase similarly for both groups. The effect of session was significant, *F*(5, 145) = 3.01, *p* = .032, η_p_^2^ = .09, 90% CI = [.01, .15], but the effect of group and group by session interaction were not significant (smallest *p* = .2).

**Figure 11 fig11:**
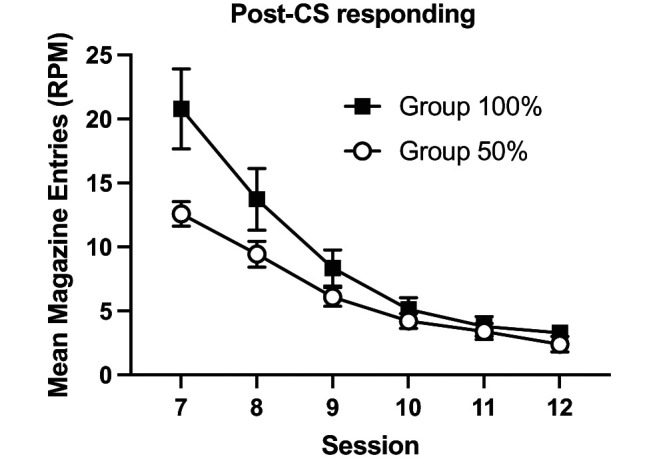
Post-CS Responding to the Nonreinforced Cue B During the Extinction Phase of Experiment 3 *Note.* Group 100% initially responded at a higher rate than Group 50%. Error bars indicate ±*SEM*. CS = conditioned stimulus; RPM = rate per minute; *SEM* = standard error of the mean.

The response rates in the 10 s period immediately after the presentation of B, the extinction cue, are shown in Figure 11. The 100% group showed greater responding than Group 50%. This effect reduced over the course of extinction as responding reduced overall for both groups. A 2 (group) × 6 (session) ANOVA showed a significant effect of session, *F*(5, 145) = 60.29, *p* < .001, η_p_^2^ = .68, 90% CI = [.59, .72]. The effect of group failed to reach significance, *F*(1, 29) = 3.91, *p* = .06, but the group by session interaction was significant, *F*(5, 145) = 4.63, *p* = .015, η_p_^2^ = .14, 90% CI = [.04, .20]. Simple main effects analysis of the group by session interaction revealed that Group 100% responded significantly more than Group 50% on the first session of extinction, *F*(1, 29) = 6.65, *p* = .015, but not thereafter, *p* > .1.[Fig fig11]

In contrast to the findings in rats (Chan & Harris, 2019), the probability of reinforcement per trial failed to affect extinction learning. Similar to the results of Experiment 2, mice responded more in the post-CS period after the previously 100% reinforced cue than after the 50% reinforced cue. Therefore, mice had learnt information about the probability of reinforcement per trial, but it did not affect the rate of extinction learning.

## Experiment 4

Experiment 3 failed to find an effect of probability of reinforcement on extinction learning. Experiment 4 was similar to Experiment 3, but the manipulation of probability of reinforcement per trial was within subjects rather than between subjects (see Table 4). Although within-subjects procedures have the potential to increase sensitivity of measures, they introduce the potential that the generalization of learning between cues may diminish effects on responding that would otherwise occur. In order to reduce the effect of generalization between cues, the 50% reinforced cue was of a different modality than the 100% reinforced cue. Furthermore, each animal was trained with two 50% and two 100% reinforced cues and one cue of each type was nonreinforced in the extinction phase, while the other cues continued to be reinforced. This design allows the detection of any generalization effect based on reinforcement contingency (i.e., 50% or 100%) that might reduce the detection of a PREE.[Table tbl4]

### Method

#### Subjects and Apparatus

Sixteen naïve female C57BL/6J mice (Charles River UK Ltd), approximately 14 weeks old at the start of testing, with a mean free-feeding weight of 22.1 g (range = 19.6–24.6) were used. All other details were the same as Experiment 1.

#### Procedure

In Stage 1, mice received 12 sessions of training with four different cues. Cues A and X were a variable duration of 10 s and were reinforced on a random 50% of trials. Cues B and Y were a variable duration of 20 s and reinforced on 100% of trials. There were six trials of A and X each per session and three of B and Y per session. The difference in the number of trials resulted in all cues having the same cumulative exposure per session and the same number of pairings with reinforcement. Trial types were presented in a random order with the constraint that for every block of six trials there were two trials of A and X each and one of B and Y each. The intertrial interval was a fixed duration of 120 s. Sessions lasted 40 min. For half of the mice, Cues A and X were auditory (noise and clicker) and B and Y were visual (house light and alternating left and right flashing LEDs, 0.5 s on, 0.5 s off). For the remaining mice, A and X were visual cues and B and Y were auditory cues. Within each subgroup, the allocation of stimuli to cues was counterbalanced in 2 by 2 manner.

In Stage 2, mice continued to receive presentations of Cue A reinforced on 50% of trials, six trials per session and Cue B reinforced on 100% of trials, three trials per session. Mice also received nonreinforced presentations of Cues X and Y on separate trials. Half of the mice received six presentations of X and Y per session, and each trial was a fixed duration of 10 s. The remaining half of mice received three presentations of X and Y, and each trial was a fixed duration of 20 s. Trial types were presented in a random order with the constraint that for the mice for which X and Y were 10 s long, there was two presentations of A, X, and Y, and one of B every block of seven trials. For the mice for which X and Y were 20 s long, each block of five trials consisted of one each of B, X, and Y and two of A. Mice received 14 sessions in Stage 2. The intertrial interval was a fixed duration of 120 s. This results in sessions lasting for 46 min for mice that received six 10 s presentations of the nonreinforced cues and 34 min for mice that received three 20 s presentations of the nonreinforced cues.

#### Data Analysis

The design of the within-subjects manipulation resulted in counterbalancing of the modality of the 50% and 100% reinforced cues across mice. We have previously found that mice show greater magazine entries for auditory cues paired with food than visual cues (Sanderson et al., 2016). In order to assess the effect of the key manipulations independent of the variance caused by this counterbalancing factor, the counterbalancing of modality was included as a factor in the analysis of response rates, but the main effect and any interactions with other factors were ignored.

For the results of the extinction phase, preliminary analysis of the effect of the number of extinction trials per session (i.e., three 20-s trials or six 10-s trials) showed that trial number had little effect (*F* < 1). For ease of exposition, the results are shown collapsed across this factor and the factor is not included in the reported analysis.

### Results and Discussion

#### Stage 1

**Figure 12 fig12:**
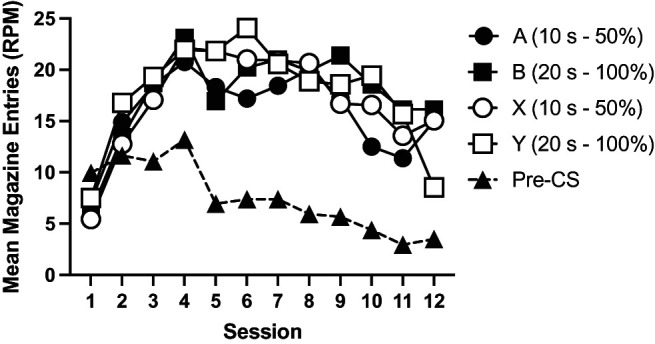
Acquisition of Conditioned Responding in Stage 1, Experiment 4 *Note.* For each cue, the durations and percentages indicate the mean trial duration and probability of reinforcement per trial. RPM = rate per minute; A = Cue A; B = Cue B; X = Cue X; Cue Y; CS = conditioned stimulus.

Mice acquired responding to the 50% reinforced cues in a similar manner as the 100% reinforced cues (see Figure 12). The data were analyzed, collapsing over the two cues that were 50% reinforced and the two cues that were 100% reinforced, using a 2 (probability: 50%, 100%) by 12 (session) by 2 (modality counterbalancing factor) ANOVA. There was a significant effect of session, *F*(11, 154) = 5.41, *p* = .002, η_p_^2^ = .28, 90% CI = [.14, .32]. The effect of partial reinforcement was not significant, *F*(1, 14) = 3.45, *p* = .08, and did not significantly interact with session, *F*(11, 154) = 1.48, *p* = .14.[Fig fig12]

A Bayesian ANOVA was conducted to test evidence for the null hypothesis that there was no difference in the acquisition of responding to partially and continuously reinforced cues. For the effect of cue, BF_incl_ = 0.39. For the cue by session interaction, BF_incl_ = 0.13. Therefore, the results provided at least 2.56 times greater evidence for the null hypothesis over the alternative hypothesis.

Additional analyses were conducted comparing response rates between cues under matched conditions (see the online supplemental materials). The analyses compared responding across similar durations within the first trial of each trial type across sessions in the latter half of training (see Supplemental Figure 6 in the online supplemental materials). There were no significant differences between partially and continuously reinforced cues. Pre-CS responses reduced over training. There was a significant effect of session, *F*(11, 154) = 11.21, *p* < .001, η_p_^2^ = .44, 90% CI = [.31, .49].

#### Extinction Stage

**Figure 13 fig13:**
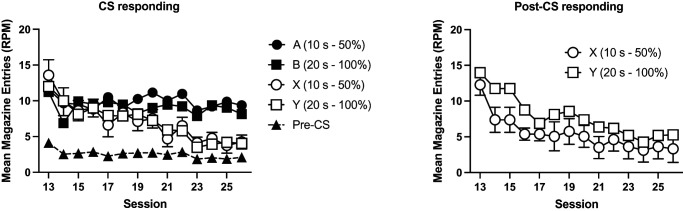
Extinction Phase of Experiment 4 *Note.* The left panel shows the selective extinction of conditioned responding to Cues X and Y over the course of training. The right panel shows the post-CS responding to the nonreinforced Cues X and Y. Mice showed greater responding to the previously 100% reinforced cue than the 50% cue. For both panels, the error bars indicate ±*SEM* within-subject difference between Cues X and Y. CS = conditioned stimulus; RPM = rate per minute; A = Cue A; B = Cue B; X = Cue X; Cue Y; *SEM* = standard error of the mean.

The results of the extinction stage are shown in Figure 13. The results were analyzed with a 2 (extinction: nonreinforced/reinforced) by 2 (probability: 50%/100%) by 2 (modality counterbalancing factor) by 14 (session) ANOVA. Mice responded significantly less to the nonreinforced cues than reinforced cues, *F*(1, 14) = 9.14 *p* = .01, η_p_^2^ = .40, 90% CI = [.07, .60]. There was a significant effect of session, *F*(13, 182) = 2.82, *p* = .049, η_p_^2^ = .17, 90% CI = [.04, .19], which significantly interacted with reinforcement, *F*(13, 182) = 5.63, *p* < .001, η_p_^2^ = .29, 90% CI = [.15, .32]. The effect of probability, the probability by reinforcement interaction, and the three-way reinforcement by session by probability interactions were not significant, *F* < 1. The remaining main effects and interactions were not significant, *p* > .3. Simple main effects analysis of the reinforcement by session interaction showed that responding to nonreinforced cues (X and Y) was significantly lower than to reinforced cues (A and B) on Sessions 17 (fifth extinction session) and 21–26 (last six extinction sessions), largest *p* = .02, but not on the remaining sessions, smallest *p* = .06.[Fig fig13]

A Bayesian ANOVA was conducted in order to assess the strength of evidence for the null hypothesis compared to the alternative hypothesis. To simplify the analysis, the effect of extinction was reduced to the difference between response rates to reinforced and nonreinforced cues such that a cue (probability: 50% vs. 100%) by session ANOVA was conducted. For the effect of cue, BF_incl_ = 0.33 and for the cue by session interaction, BF_incl_ = 0.01. Therefore, evidence for the null hypothesis was at least 3 times greater than for the alternative hypothesis. Pre-CS response rates were stable over extinction training. There was no significant effect of session, *F*(13, 182) = 1.64, *p* = .17.

Mice showed greater post-CS responding to the previously reinforced 100% cue than the 50% reinforced (see Figure 13, right panel). Responding was analyzed with a 2 (cue: 50%, 100%) by 2 (modality counterbalancing factor) by 14 (session) ANOVA. There was a significant effect of cue, *F*(1, 14) = 7.46, *p* = .016, η_p_^2^ = .35, 90% CI = [.04, .56], significant effect of session, *F*(13, 182) = 5.95, *p* < .001, η_p_^2^ = .30, 90% CI = [.16, .34], but no significant cue by session interaction, *F* < 1.

The results were similar to those of Experiment 3. The within-subjects procedure failed to provide evidence of a PREE, but post-CS responding demonstrated that mice were sensitive to the manipulation of probability of reinforcement per trial. In comparison to Experiment 3, the extinction of responding took longer to emerge over the course of training. This may have been because of the increased generalization between cues. Whereas mice learnt about only two cues in Experiment 3, mice learnt about four cues in Experiment 4 and in the extinction phase had to discriminate between stimuli within two different modalities. Despite the seemingly different rates of extinction between the two experiments neither provided evidence of a PREE. The discussion of the potential differences between the results of Experiment 3 and 4, and those in rats (Chan & Harris, 2019) are saved for the General Discussion section.

## General Discussion

The probability of reinforcement per trial failed to affect overexpectation and extinction. Furthermore, in each experiment, it failed to affect acquisition of conditioned responding and, in Experiments 1 and 2, summation of conditioned responding. The lack of effect is consistent with the finding that cumulative rate of reinforcement rather than probability of reinforcement per trial is the key determinant of conditioned responding (Harris et al., 2015). In contrast to the lack of effect on conditioned responding during the presentation of CSs, the probability of reinforcement did affect post-CS responding on nonreinforced trials. Mice showed greater responding at the termination of cues that were previously 100% reinforced than for cues that were previously 50% reinforced. The dissociation between CS and post-CS responding suggests that mice learn about the probability of reinforcement per trial as well rate of reinforcement, but it does not affect responding to a CS prior to the expected time of the US.

The results of Experiments 1 and 2 lead to several conclusions about the cause of overexpectation. The first concerns the role of summation. The Rescorla and Wagner (1972) model proposes that overexpectation is the result of summation of the associative strength of cues when presented in compound. In Experiments 1 and 2, the extent of summation was similar for cues that differed in probability of reinforcement per trial but were matched for reinforcement rate. Therefore, the learning that was summed likely reflects learning of the reinforcement rate of the individual cues and not the probability of reinforcement. This builds on work by Andrew and Harris (2011) that showed that in a summation test, responding to the test compound was similar to that for a cue or compound that had a reinforcement rate that was sum of the reinforcement rate of the individual cues in the test compound. This was true regardless of whether reinforcement rate was manipulated by CS duration or probability of reinforcement per trial. Therefore, this may suggest that summation not only reflects learning about reinforcement rates but it also reflects specifically summation of expected reinforcement rate. The second conclusion is that the learning process that determines the reduction of conditioned responding also reflects learning of reinforcement rate rather than probability of reinforcement per trial. Therefore, the cause of summation and the subsequent overexpectation reflects a common process.

The results are similar to a certain extent to findings in rats. Similar to Experiments 2–4, Harris and Kwok (2018) found that post-CS responding was a function of the probability of reinforcement per trial in cues matched for reinforcement rate. The difference occurred despite similar response rates during the CS presentation. Therefore, learning in mice and rats is sensitive to both probability of reinforcement per trial and reinforcement rate over cumulative exposure. The dissociation suggests that there are separate learning mechanisms that update either continuously over time, in order to learn about reinforcement rate, or episodically, to learn about probability of reinforcement per trial. The fact that animals are sensitive to trial-based information suggests that animals separate experience into discrete events.

The results do, however, differ distinctly from findings in rats. Harris and colleagues have shown that extinction learning in rats is sensitive to probability of reinforcement per trial under conditions in which reinforcement rate is controlled (Chan & Harris, 2017, 2019; Harris, 2024; Harris, Kwok, & Gottlieb, 2019; Harris, Seet, & Kwok, 2019; Jiao & Harris, 2024; Norton & Harris, 2022). There was no evidence that this was the case for mice. Experiment 3 used a between-subjects design and despite differences in post-CS responding, responding during the CSs extinguished at a similar rate over sessions. Experiment 4 replicated these findings but used a within-subjects design. Not only is this a failure to observe an effect of probability of reinforcement per trial on extinction under conditions in which reinforcement rate is matched between the cues, it is a failure to observe a PREE generally. Failures to observe a PREE in Pavlovian procedures (e.g., Pearce et al., 1997) have been attributed to potential generalization between cues in within-subjects procedures such that a difference in extinction is not observed or to extinction of magazine approach behavior independent of extinction of the CS, which may have masked a PREE. These factors are unlikely to contribute to the lack of effect in the present experiments because the inclusion of a reinforced cue (Experiment 3) or cues (Experiment 4) during the extinction phase maintained reinforcement of the magazine and reduced generalization between cues because mice learnt to discriminate between reinforced and nonreinforced, extinguished cues. Indeed, in Experiment 4, in which a within-subjects manipulation of partial reinforcement was used, one cue from each modality (visual, auditory) was extinguished such that discrimination between cues could not be achieved by modality alone and required mice to discriminate between individual cues on the basis of their reinforcement contingency. Furthermore, the differences in post-CS responding indicate that mice discriminated between cues that extinguished at similar rates. This also suggests that is unlikely that the overall change in the amount of reinforcement between the acquisition and extinction phases resulted in increased generalization between the cues.

A PREE has been observed in mice under conditions that have not controlled for reinforcement rate. For example, it has been observed in the extinction of the speed of traversing a runway for appetitive reinforcement (Lee et al., 1974; Wong et al., 1971) and in extinction of Pavlovian conditioned freezing to shock (Huh et al., 2009). Therefore, it is unlikely that the absence of the effect in Experiments 3 and 4 is simply because of an inability to observe an effect of partial reinforcement on extinction in mice generally. A recent study by Mallea et al. (2024) used an appetitive Pavlovian conditioning procedure in mice that was similar to those used in the current study. From Experiment 2 of their study, Mallea et al. (2024) concluded that partial reinforcement, when reinforcement rate is controlled, does result in a PREE. This conclusion, however, is potentially undermined by initial differences in responding as a result of partial and continuous reinforcement prior to extinction. Thus, mice respond more for a continuously reinforced 24 s cue than a 12 s cue reinforced on 50% of trials. While responding extinguished from an initial higher rate for the continuously reinforced group than the partially reinforced group, it did not come to be extinguished below the rate of responding for the partially reinforced group. Therefore, it is hard to come to firm conclusions about the rate of extinction when it starts from different initial levels of responding. The study by Mallea et al. (2024) did find, however, that mice responded less when reinforced on 50% of trials than on 100% of trials despite matching of overall reinforcement rates. The effect emerged during training as a consequence of mice reducing responding to the 50% reinforced cue compared to the 100% cue. This finding is at odds with the absence of an effect that was observed in the acquisition data across Experiments 1–4 and with other findings in mice (Austen et al., 2021) and rats (Harris et al., 2015). While there are a number of procedural differences between the studies, it is not clear what the cause of the discrepancy in findings is between the Mallea et al. (2024) study and the present experiments.

While the reasons for why a PREE was not observed in mice are not clear, the dissociation between the sensitivity of post-CS responding and extinction learning demonstrates that the expectation of the US that determines post-CS responding does not determine subsequent extinction of conditioned responding during the CS. Harris (2019) suggested that trial-based learning is responsible for extinction rate because it is only at the end of a trial that an animal can determine that the US has not occurred. Thus, learning about nonreinforcement reflects trial-based probability learning because the termination of trial is an event that determines the US has not and will not occur. This results in partially reinforced cues requiring more nonreinforced trials for extinction to occur than continuously reinforced cues. The present results demonstrate that mice are sensitive to trial-based information, but it does not determine the rate of extinction. Furthermore, a similar dissociation was observed in Experiment 2, demonstrating that, despite an effect on post-CS responding, trial-based information does not determine the extent of overexpectation. Thus, although mice responded at a significantly higher rate after a compound of two individually 100% reinforced cues compared to two 50% reinforced cues, the extent of overexpectation was similar. While the dissociation in mice does not mean that the cause of the post-CS responding effect is not the cause of the PREE in rats, it does demonstrate that the cause of the post-CS responding effect is not sufficient for the PREE.

Harris and Kwok (2018) demonstrated that the effect of probability of reinforcement per trial on post-CS responding was not simply because of learning that the offset of the cue predicted the probability of reinforcement. The post-CS responding effect was still present when the US occurred at a random interval within the CS rather than at the termination of the CS. Therefore, the offset of the CS was not a cue for the US. This finding is important because it would be expected that with variable duration CSs the CS offset is more informative than the CS onset about the occurrence of reinforcement. Therefore, despite this consequence of trial duration variability, the effect nonetheless depended on the probability of reinforcement per trial signaled by the presence of the CS prior to the termination of the trial. In the present experiments, the US always occurred at the termination of a CS. We cannot rule out the possibility that mice had learnt that the offset of the cue signaled the probability of reinforcement. Indeed, given this possibility, an account of the dissociation observed in mice can be made without appealing to independent trial-based and time-based learning mechanisms. If it is assumed that the immediate trace of the CS is paired with the US (e.g., the trace CS undergoes simultaneous or backward conditioning), then the strength of post-CS responding may reflect the reinforcement rate of the trace CS. For cues that differ in probability of reinforcement but are matched for reinforcement rate by virtue of differences in CS duration, the reinforcement rate of the trace of a partially reinforced cue will be lower than the reinforcement rate of the trace of a continuously reinforced cue. For this account to be plausible it would have to be assumed that the trace of a long duration cue has a similar saliency and decays at a similar rate as the trace of a short duration cue. While this is possible, it may be unlikely given that it has been proposed that the reduction of conditioned responding to a long CS compared to a short CS is the consequence of the salience of a CS decreasing over the duration of its presentation (Harris & Bouton, 2020) as a consequence of short-term habituation (Brandon et al., 2003; Vogel et al., 2019; Wagner, 1981). Further work is needed to conclude that the post-CS responding in mice is specifically a function of probability of reinforcement independent of CS duration. However, the dissociations present in Experiments 2–4 show that mice had clearly learnt information about the cues that differed in probability of reinforcement but it affected post-CS responding only.

It is possible that the difference between CS responding and post-CS responding simply reflects that post-CS responding is a more sensitive measure of learning about the CS than CS responding. This is unlikely to be the case for magazine approach behavior in rats because the factors that affect CS and post-CS responding can be doubly dissociated (Harris & Kwok, 2018). Thus, CSs that are matched for reinforcement rate but differ in probability of reinforcement per trial elicit similar levels of conditioned responding during the CS but different levels after the CS. CSs that are matched for probability of reinforcement per trial but differ in reinforcement rate elicit different levels of conditioned responding during the CS but similar levels after the CS. In mice, magazine approach behavior during a CS is sensitive to reinforcement rate, independent of whether it is manipulated by CS duration or probability of reinforcement (Austen & Sanderson, 2019; Austen et al., 2022; Strickland et al., 2024), but as shown in the present experiments, when reinforcement rate is controlled, probability of reinforcement fails to affect performance. If post-CS responding is simply a more sensitive measure of learning about the CS than responding during the CS, then this would make the results of Experiments 3 and 4 even more surprising because rather than simply a failure to observe the PREE, it would suggest that the opposite effect was found (i.e., slower extinction for a continuously reinforced CS compared to a partially reinforced CS). This is unlikely given the observations of a PREE in mice in other procedures (Huh et al., 2009; Lee et al., 1974; Wong et al., 1971). Therefore, the results suggest that CS responding and post-CS responding reflect different forms of learning.

Across the experiments, the probability of reinforcement per trial was manipulated under conditions in which the reinforcement rate was controlled. The absence of an effect of probability of reinforcement on acquisition is consistent with other findings (Austen et al., 2021; Harris et al., 2015). Given the positive evidence of an effect of reinforcement rate on acquisition (Austen et al., 2021; Harris et al., 2015), this suggests that reinforcement rate is the primary determinant of conditioned responding and not probability of reinforcement. The failure to find an effect of probability of reinforcement on overexpectation and extinction may also suggest that reinforcement rate is more important; however, the experiments, although they included control procedures for overexpectation and extinction, did not include manipulations of reinforcement rate that held probability of reinforcement constant. It seems reasonable to assume that in the absence of an effect of probability of reinforcement per trial, overexpectation and extinction must rely on reinforcement rate but a positive demonstration would make this conclusion compelling. At the least, evidence from other effects that assess manipulations of prediction error and/or summation in compound conditioning procedures have provided evidence that reinforcement rate and not probability of reinforcement is sufficient to result in learning. Harris et al. (2012) found that in a summation test in rats, summation of responding reflection summation of reinforcement rates regardless of whether reinforcement rate was manipulated by trial duration or probability of reinforcement per trial. Similarly, conditioned inhibition was acquired for cues that signaled a decrease in reinforcement rate regardless of whether rate was manipulated by trial duration or probability of reinforcement per trial (Harris et al., 2014). For overexpectation, it would be predicted that the extent of overexpectation would depend on the summation of reinforcement rates and the degree to which the summation of associative strength exceeded the maximum associative strength supported by the US (lambda). For example, for a cue that signals a high reinforcement rate, overexpectation would be greater when that cue was reinforced in compound with another high reinforcement rate cue than with a lower reinforcement rate cue.

In conclusion, it remains to be seen whether overexpectation and extinction are determined by similar factors in rats. In mice, however, the present results suggest that they share a common a process that may reflect learning about reinforcement rate but not probability of reinforcement per trial. This suggests that learning that determines anticipatory responding is continuously updated over time rather than in a trial-dependent manner.

## Supplementary Material

10.1037/xan0000396.supp

## Figures and Tables

**Table 1 tbl1:** Design of Experiment 1

Group	Stage 1	Stage 2	Test
50%	A—10 s, 50%; B—20 s, 100%; X—10 s, 50%; Y—20 s, 0%	AX—20 s, 100%; BY—20 s, 100%	A—20 s, 0%; B—20 s, 0%; AX—20 s, 100%; BY—20 s, 100%
100%	A—20 s, 100%; B—20 s, 100%; X—20 s, 100%; Y—20 s, 0%		
*Note.* The letters A, B, X, and Y denote cues. The durations denote the average trial duration of the cue (see the Method for details). The percentages indicate the probability of reinforcement per trial. Cues that were on average 10 s were presented twice as often as 20 s cues.			

**Table 2 tbl2:** Design of Experiments 2a and 2b

Experiment	Group	Stage 1	Stage 2	Test
Experiment 2a	50%	A—10 s, 50%; B—10 s, 50%; X—10 s, 50%; Y—10 s, 0%	AX—10 s, 50%; BY—10 s, 50%	A—10 s, 0%; B—10 s, 0%; AX—10 s, 50%; BY—10 s, 50%
	100%	A—20 s, 100%; B—10 s, 50%; X—20 s, 100%; Y—10 s, 0%		
Experiment 2b	50%	A—10 s, 50%; B—10 s, 50%; X—10 s, 50%; Z—10 s, 0%		
	100%	A—20 s, 100%; B—10 s, 50%; X—20 s, 100%; Z—10 s, 0%		
*Note.* The letters A, B, X, and Y denote cues. The durations denote the average trial duration of the cue (see the Method for details). The percentages indicate the probability of reinforcement per trial. Cues that were on average 10 s were presented twice as often as 20 s cues. The main difference between experiments was that Cue Y was a novel cue at the beginning of Stage 2 in Experiment 2b, but a familiar cue in Experiment 2a.				

**Table 3 tbl3:** Design of Experiment 3

Group	Cue A treatment (%)	Stage 1	Stage 2
Continuous	100	A—20 s, 100%; B—20 s, 100%	A—20 s, 100%; B—10 s, 0%
Partial		A—20 s, 100%; B—10 s, 50%	
Continuous	50	A—10 s, 50%; B—20 s, 100%	A—10 s, 50%; B—10 s, 0%
Partial		A—10 s, 50%; B—10 s, 50%	
*Note.* The letters A and X denote cues. The durations denote the average trial duration of the cue (see the Method for details). The percentages indicate the probability of reinforcement per trial. Cues that were on average 10 s were presented twice as often as 20 s cues.			

**Table 4 tbl4:** Design of Experiment 4

Group	Cue A treatment (%)	Stage 1	Stage 2
Continuous	100	A—20 s, 100%; B—20 s, 100%	A—20 s, 100%; B—10 s, 0%
Partial		A—20 s, 100%; B—10 s, 50%	
Continuous	50	A—10 s, 50%; B—20 s, 100%	A—10 s, 50%; B—10 s, 0%
Partial		A—10 s, 50%; B—10 s, 50%	
*Note.* The letters A and X denote cues. The durations denote the average trial duration of the cue (see the Method for details). The percentages indicate the probability of reinforcement per trial. Cues that were on average 10 s were presented twice as often as 20 s cues.
