# Supplementary material for: Probability and Rate of Reinforcement in Negative Prediction Error Learning
Source: J Exp Psychol Anim Learn Cogn. 2025 May 12;51(3):126–46. doi: 10.1037/xan0000396 (PMC12224701; doi:10.1037/xan0000396)
Supplement: Supplementary file 1 [file XAN-2024-0325_Supplemental_Material.docx]

Supplemental Material

**Probability and rate of reinforcement in negative prediction error learning**

David J. Sanderson, Joseph M. Austen, Anthony McGregor and Jasmin A. Strickland

**Supplemental Results**

**Experiment 2**

Independent analyses of the test data (responding to cues A and B) for Experiment 2a and 2b:

*Experiment 2a*

Effect of cue (A vs. B): F(1,30) = 3.17, p = 0.085. Effect of group: F(1,30) = 1.33, p = 0.26. Cue by group interaction: F < 1.

*Experiment 2b*

Effect of cue (A vs. B): F(1,30) = 4.14, p = 0.051. Effect of group: F < 1. Cue by group interaction: F < 1.

The trial-by-trial data collapsed across experiments for group 50% and group 100% are shown in Supplement Figure 1. A 20 (trial) by 2 (group: 50%, 100%) by 2 (cue: A, B) by 2 (experiment) ANOVA showed a significant effect of cue, F(1,60) = 7.16, p = 0.01, η_p_^2^ = .11, 90% CI [.02, .23], and a significant effect of trial, F(19,1140) = 6.21, p < 0.001, η_p_^2^ = .09, 90% CI [.06, .11]. The cue by trial interaction was not significant, F < 1, but the linear trend for the interaction approached significance, F(1,60) = 3.37, p = 0.07. The cue by group interaction was not significant, F(1,60) = 1.07, p = 0.31, and there was no significant cue by group by trial three-way interaction, F < 1. Mice in Experiment 2a responded at a higher rate than mice in Experiment 2b, F(1,60) = 4.79, p = 0.03, η_p_^2^ = .07, 90% CI [.003, .19]. There were no other significant main effects or interactions of factors (smallest p-value = 0.14).

**

*Supplemental Figure 1. Responding as a function of test trial in the overexpectation test, Experiment 2a and 2b. Mean magazine entries are shown as rate per minute (RPM). The error bars for cue A indicate ±standard error of the mean within-subject difference between cues A and B.*

**Experiment 3**

Analysis of the acquisition and extinction phases of Experiment 3 that include the training regime of cue A, the cue that was consistently reinforced across both phases (see Supplemental Figure 2). For approximately half the mice within each group, cue A was a variable duration 10 s cue that was reinforced on 50% of trials or a variable 20 s cue that was reinforced on 100% of trials.

Acquisition: Effect of session: F(5, 135) = 63.5, p < 0.001. All of other main effects and interactions, p-values > 0.11.

Extinction: Effect of cue: F(1,27) = 75.7, p < 0.001. Effect of session: F(5, 135) = 9.62, p < 0.001. Cue by session interaction: F(5, 135) = 16.9, p < 0.001. Effect of cue A reinforcement contingency: F(1,27) = 5.63, p = 0.025. All other main effects and interactions, p-values ≥ 0.09.

*Supplemental Figure 2. Experiment 3, acquisition (sessions 1-6) and extinction (sessions 7-12) phases. The results are shown for the 100% (continuously reinforced) group (top panels) and the 50% (partially reinforced) group (bottom panels) broken down by the treatment of cue A. The left panels show the results for the mice trained with cue A as a variable 10 s cue that was reinforced on 50% of trial. The right panels show the results for the mice trained with cue A as a variable 20 s cue that was reinforced on 100% of trials.*

**Analyses of the effect of partial reinforcement on response rates under matched test conditions for Experiments 1-4**

Comparison of the rate of responding for 50% reinforced 10 s cues and 100% reinforced 20 s cues is potentially confounded by the measurement of responses over different trial durations. There is typically a delay in initiating responding within a trial regardless of the duration of the cue. Consequently, measurement of response rates will more frequently occur within these initial periods for short duration cues than for long duration cues. Another potential confound is that 50% reinforced cues, although matched for total exposure, occur more frequently within a session than 100% reinforced cues. Therefore, after the first trial each trial type within a session the frequency and average inter-trial interval of the cues will differ.

In order to assess response rates for cues that differ in probability of reinforcement per trial but are matched reinforcement rate it is necessary to assess to performance under similar test conditions. This was achieved by assessing performance on the first trial of each trial type within a session across matched trial durations. Further details about the analyses for specific experiments is provided below.

*Experiment 1*

Response rates were analysed for the first trial of each trial type across sessions 7-12, the latter half of acquisition training, in which group-level response rates were maximal over sessions. The mean response rate per second was calculated taking into account that the trial durations differed across trial types and sessions (e.g., responding across seconds 1-2 of the trial was always measured because each trial was at least 2 s long but responding across later periods was less often measured). Thus, the mean response rate per second reflected the response rate independent of the frequency of sampling the specific time period. Given the variation in duration of trials, it was possible to compare responding across a time period of 12 s for all cues (see Supplemental Figure 3).

An ANOVA, using the Greenhouse-Giesser correction, revealed a significant effect of cue, F(3,90) = 3.13, p < 0.001, and time bin (s), F(11,330) = 6.57, p < 0.001, and a significant interaction of those factors, F(33,990) = 1.95, p = 0.001. The effect of group and all interactions including the effect of group were not significant, p-values > 0.18. An additional ANOVA that just compared responding for the reinforced cues failed to show a significant effect of cue, F(2,60) = 1.60, p = 0.22, nor any significant interactions that included the effect of cue and group, F-values < 1.

A Bayesian analysis performed in JASP using the default priors was conducted for the response rates for reinforced cues. Bayes factors for main effects or interactions reflect comparison of models that include the main effect or interaction with similar models that exclude the main effect or interaction. For the effect of cue, BF_incl_ = 0.27. For the cue x group interaction, BF_incl_ = 0.13. For the cue x group x bin interaction, BF_incl_ = 0.001.

*Supplemental Figure 3. Mean rates of responding in Experiment 1 for up to the first 12 s of the first trials of each trial type of a session, collapsed across sessions 7-12.*

*Experiment 2*

Responding was analysed in a similar manner as Experiment 1. The sampled time period that was common to all four cues was 12 s and is shown in Supplemental Figure 4. ANOVA of responding to all four cues showed a significant effect of cue, F(3,180) = 31.52, p < 0.001, time bin (s), F(11,660) = 8.02, p < 0.001 and replication (Experiment 2a vs. Experiment 2b), F(1,60) = 11.95, p < 0.001. There was a significant cue by bin interaction, F(33,1980) = 1.74, p = 0.03. No other main effects or interactions were significant, p-values > 0.26. An ANOVA comparing response rates of just the three reinforced cues failed to show a significant effect of cue, F < 1, or interactions including the factors of cue and group, p-values > 0.4.

A Bayesian analysis similar to the one for Experiment 1 was conducted. For the effect of cue, BF_incl_ = 0.06. For the cue by group interaction, BF_incl_ = 0.05. For the cue by bin by group interaction, BF_incl_ = 0.0005.

**

*Supplemental Figure 4. Mean rates of responding in Experiment 2 for up to the first 12 s of the first trials of each trial type of a session, collapsed across sessions 7-12.*

*Experiment 3*

Due to the manner in which the data was collected by the programme for Experiment 3 it was not possible to identify the order in which reinforced and nonreinforced trials were presented with a session. Therefore, this did not allow analysis of the first trial of each trial type per session during the latter half of acquisition as was conducted for Experiments 1 and 2. Instead, performance was analysed for the first trial of the extinction cue during the first session of the extinction stage of the experiment. This first trial allowed assessment of responding before exposure to the extinction reinforcement contingency, and, therefore, allows a matched test of learning prior to extinction. The trial duration of the first trial was 9 s. Response rates are shown in Supplemental Figure 5.

An ANOVA revealed a significant effect of time bin (s), F(8,232) = 3.46, p = 0.003. There was no significant effect of group, F < 1 and no significant interaction of factors, F(8,232) = 1.23, p = 0.30.

A Bayesian ANOVA was conducted. For the effect of group, BF_incl_ = 0.32. For the interaction of time bin by group, BF_incl_ = 0.15.

*Supplemental Figure 5. Mean rates of responding in Experiment 3 for the 9 s duration of the first trial of the extinction cue in the first extinction session (session 7).*

*Experiment 4*

The sampled time period that was common to all four cues across sessions 7-12 of acquisition was 12 s and is shown in Supplemental Figure 6. An ANOVA was conducted collapsing across the extinction and non-extinction cues that were reinforced either 50% of 100%. There was no significant effect of cue (i.e., effect of partial reinforcement), F < 1. There was a significant effect of time bin (s), F(11,154) = 4.90, p < 0.001. The cue by bin interaction was not significant, F(11,154) = 1.96, p < 0.08.

A Bayesian analysis was conducted. For the effect of cue, BF_incl_ = 0.28. For the cue by bin interaction, BF_incl_ = 3.90.

**

*Supplemental Figure 6. Mean rates of responding in Experiment 4 for up to the first 12 s of the first trials of each trial type of a session, collapsed across sessions 7-12.*
